# Mechanism and linkage specificities of the dual retaining β-Kdo glycosyltransferase modules of KpsC from bacterial capsule biosynthesis

**DOI:** 10.1016/j.jbc.2023.104609

**Published:** 2023-03-15

**Authors:** Liam Doyle, Olga G. Ovchinnikova, Bo-Shun Huang, Taylor J.B. Forrester, Todd L. Lowary, Matthew S. Kimber, Chris Whitfield

**Affiliations:** 1 Department of Molecular and Cellular Biology, University of Guelph, Guelph, Ontario, Canada; 2 Department of Chemistry, University of Alberta, Edmonton, Alberta, Canada; 3 Institute of Biological Chemistry, Academia Sinica, Taipei, Taiwan; 4 Institute of Biochemical Sciences, National Taiwan University, Taipei, Taiwan

**Keywords:** *Escherichia coli*, cell surface, capsular polysaccharide, glycolipid, biosynthesis, 3-deoxy-β-D-manno-oct-2-ulosonic acid, glycosyltransferase, KpsC, enzyme structure, enzyme mechanism

## Abstract

KpsC is a dual-module glycosyltransferase (GT) essential for “group 2” capsular polysaccharide biosynthesis in *Escherichia coli* and other Gram-negative pathogens. Capsules are vital virulence determinants in high-profile pathogens, making KpsC a viable target for intervention with small-molecule therapeutic inhibitors. Inhibitor development can be facilitated by understanding the mechanism of the target enzyme. Two separate GT modules in KpsC transfer 3-deoxy-β-d-*manno*-oct-2-ulosonic acid (β-Kdo) from cytidine-5′-monophospho-β-Kdo donor to a glycolipid acceptor. The N-terminal and C-terminal modules add alternating Kdo residues with β-(2→4) and β-(2→7) linkages, respectively, generating a conserved oligosaccharide core that is further glycosylated to produce diverse capsule structures. KpsC is a retaining GT, which retains the donor anomeric carbon stereochemistry. Retaining GTs typically use an S_*N*_i (substitution nucleophilic internal return) mechanism, but recent studies with WbbB, a retaining β-Kdo GT distantly related to KpsC, strongly suggest that this enzyme uses an alternative double-displacement mechanism. Based on the formation of covalent adducts with Kdo identified here by mass spectrometry and X-ray crystallography, we determined that catalytically important active site residues are conserved in WbbB and KpsC, suggesting a shared double-displacement mechanism. Additional crystal structures and biochemical experiments revealed the acceptor binding mode of the β-(2→4)-Kdo transferase module and demonstrated that acceptor recognition (and therefore linkage specificity) is conferred solely by the N-terminal α/β domain of each GT module. Finally, an Alphafold model provided insight into organization of the modules and a C-terminal membrane-anchoring region. Altogether, we identified key structural and mechanistic elements providing a foundation for targeting KpsC.

Capsular polysaccharides are produced by many bacterial species, and they generate a protective surface layer (the capsule) that is frequently associated with evasion of host immune defenses by pathogens (reviewed in (1)). Capsular polysaccharides include a remarkably diverse range of chemical structures; for example, more than 80 distinct structures are made by *Escherichia coli* (2) and more than 90 by *Streptococcus pneumoniae* (3). Despite this variety, most capsular polysaccharides are assembled by one of the two assembly strategies. This study addresses one of those strategies, which requires a characteristic ABC transporter and is best characterized in the production of *E. coli* “group 2” capsules (reviewed in (1, 2)).

The defining feature of *E. coli* group 2 capsular polysaccharides is a conserved glycolipid terminus comprising (lyso)phosphatidylglycerol modified with a short chain of 3-deoxy-β-d-*manno*-oct-2-ulosonic acid (β-Kdo) residues (4, 5, 6) (Fig. 1*A*). Its synthesis is performed by two CMP-β-Kdo-dependent glycosyltransferases (GTs). KpsS is a monofunctional GT and initiates capsule synthesis by transferring the initial β-Kdo residue to the phospholipid (5, 7, 8). KpsC, which possesses two β-Kdo GT modules, then extends the KpsS product by assembling an oligosaccharide composed of alternating β-(2→4)- and β-(2→7)-linked Kdo residues. It sequentially transfers a β-(2→7)-linked Kdo residue, using its C-terminal GT module (KpsC-C_*Ec*_ in the *E. coli* enzyme), followed by a β-(2→4)-linked Kdo residue, transferred by the N-terminal GT module (KpsC-N_*Ec*_) (5, 7, 9) (Fig. 1*B*). Unusually, in the thermophile, *Thermosulfurimonas dismutans*, these two KpsC GT modules exist as separate polypeptides (5, 9). The resulting Kdo-glycosylated lipid appears to be conserved in almost all capsule-assembly systems which use an ABC transporter–dependent assembly strategy in Gram-negative bacteria (1). The Kdo-modified glycolipid serves as an acceptor for serotype-dependent GTs, which generate capsular polysaccharides with diverse carbohydrate structures and antigenic epitopes (1) (Fig. 1*B*). Once synthesis is completed in the cytoplasm, the polymer product is exported to the surface by a membrane-spanning translocation pathway that includes an ABC transporter and an outer membrane translocon, which are thought to be bridged by a largely periplasmic adapter protein (1, 2). Based on conserved export machinery and KpsS and KpsC enzymes, the same assembly system appears also to be used in important human pathogens including *Neisseria meningitidis*, *Haemophilus influenzae*, *Bordetella pertussis* and *bronchiseptica*, *Campylobacter jejuni* and others (7), as well as in important livestock pathogens such as *Mannheimia haemolytica* and *Actinobacillus pleuropneumoniae* (5).

**Figure 1:**
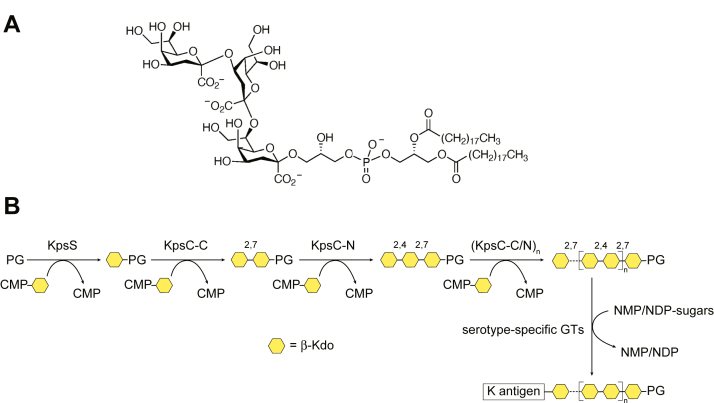
**Structure and biosynthesis of the KpsC reaction product.***Panel A* shows the structure of the phosphatidylglycerol-(Kdo)_3_ glycolipid intermediate synthesized by the combined actions of KpsS and the two GT modules in KpsC. The overall reaction scheme for the synthesis of an *Escherichia coli* group 2 capsular polysaccharide is depicted in *Panel B*, KpsS and KpsC are β-Kdo GTs, which transfer Kdo residues from CMP-β-Kdo donor to create a phosphatidylglycerol (PG)-based acceptor. KpsS adds the initial Kdo residue. This is followed by sequential addition of Kdo residues by the alternating actions of the KpsC-C and KpsC-N GT modules, to create a glycolipid acceptor containing 5 to 9 Kdo residues. The conserved KpsC reaction product is then elongated by a serotype-specific GTs to create a CPS belonging to a given K-antigen serotype. GT, glycosyltransferase.

Antibiotic regimens for many pathogens are increasingly compromised by resistance, so other approaches are needed. Vaccines provide one strategy and capsules are the targets of some existing successful vaccines, while others are currently in development (10, 11). However, developing capsule vaccines for some pathogen species is challenging because of the hypervariable carbohydrate structures and, in some instances, their mimicry of host glycans (12). Small-molecule inhibitors of capsule assembly have been proposed as one viable alternative (13). The conservation of KpsS and KpsC means that they present a therapeutic target with the potential to attenuate a broad range of Gram-negative pathogens, where capsules are required for survival in the host. A high-throughput screening assay has been developed for KpsC (14) and the present study is part of an ongoing effort to define the structure–function properties of this enzyme to strengthen the foundation for discovery of small-molecule inhibitors.

GTs catalyze the transfer of sugar residues from activated donors to acceptor substrates, forming glycosidic linkages (reviewed in (15)). In the Carbohydrate-Active Enzymes database, more than 1 million GT modules from all forms of life are currently assigned to 116 GT families (February, 2023) based on sequence and/or structural similarities (16). KpsC is the founding member of the GT107 family. Despite their incredible functional diversity, the most common GTs involved in the synthesis of bacterial surface polysaccharides are Leloir enzymes, which use nucleotide-sugar donors (such as cytidine-5′-monophospho-β-Kdo) and possess one of two folds—either the GT-A or GT-B fold (reviewed in (15)). Crystal structures of KpsC-N GT modules revealed a GT-B–like structure, where two simplified α/β Rossmann-like domains are reorganized and separated by additional inserted α-helices. Interestingly, the predominant structural differences between the KpsC-N and KpsC-C enzymes are found in the N-terminal α/β domains, which are implicated in acceptor binding in GT-B enzymes (15).

Because KpsC uses CMP-β-Kdo as its donor, and produces a β-Kdo–linked product, it is a retaining GT (5). Until recently, most mechanistically characterized retaining GTs were found to follow an S_*N*_i (substitution nucleophilic internal return) mechanism. An alternative double-displacement mechanism, analogous to the well-established mechanism of retaining glycoside hydrolases (reviewed in (17)), has been proposed for one GT6-family enzyme (18), but experimental support for broader involvement of this mechanism in retaining GTs was limited. We recently provided experimental evidence that another β-Kdo GT (WbbB, a GT99 prototype from lipopolysaccharide O-antigen biosynthesis in *Raoultella* and *Klebsiella*) uses a double-displacement mechanism (19). KpsC is a distant homolog of WbbB and conserves three critical catalytic residues found in WbbB; an aspartate that serves as the nucleophile and forms an Asp–Kdo adduct, a histidine that protonates the CMP phosphate leaving group, and a glutamate that acts as a base to activate the incoming acceptor. Earlier studies showed that KpsC variants were catalytically inactive when either of the acidic residues were changed to alanine, while variants with the histidine mutated are severely catalytically compromised (7, 11). This pointed to a mechanism similar to WbbB, but definitive evidence was lacking. The primary objective of this work was therefore to investigate the potential use of a double-displacement mechanism by KpsC. To add further insight into the structure and function of KpsC, we also examined the structural basis for acceptor binding and its role in linkage specificity in the β-(2→4) Kdo transferase module. Finally, KpsC enzymes possess a C-terminal membrane-anchoring region and we assessed how this region affects the activities of KpsC.

## Results

### KpsC-N forms covalent Kdo-adducts with Asp160 and its variants

**Figure 2:**
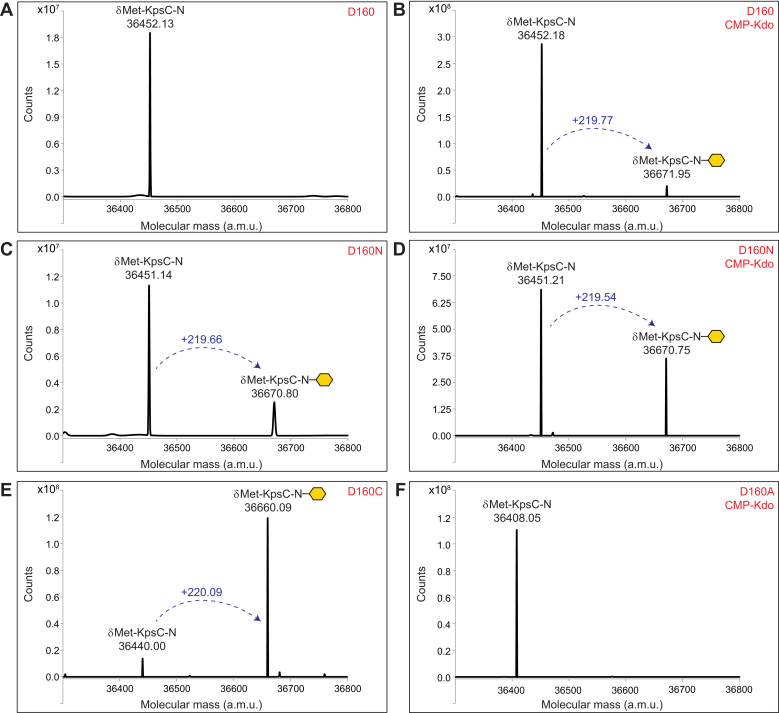
**LC-MS analysis of KpsC-N**_***Ec***_**and its active site variants to investigate the formation of covalent Kdo adducts.** KpsC-N_*Ec*_ proteins were purified from *Escherichia coli* BL21 and analyzed by LC-MS before or after incubation with a CMP-Kdo generating reaction mixture. Kdo adducts are denoted by the *yellow hexagons*. Note that the N-terminal methionine residue is absent in in all cases. The formation of a Kdo adduct should result in a 220.18 Da mass increase to the protein. *A*, the spectrum of the WT protein shows a single species corresponding to the apoprotein. *B*, the spectrum of the WT protein following incubation with a CMP-Kdo generating reaction shows formation of a second species containing a modification with Kdo. *C*, the D160N protein appears to have formed Kdo adducts *in vivo* as two species differing by 219.66 Da were observed without incubation with CMP-Kdo. *D*, following incubation with CMP-Kdo, the D160N protein appears to have an increase in the amount of Kdo-modified protein compared to *panel C*. *E*, the D160C protein appears to be almost completely modified with Kdo *in vivo* as only small amounts of unmodified protein remain. *F*, the D160A protein remains unmodified even after incubation with CMP-Kdo.

The formation of an intermediate with a covalent bond between the donor saccharide and a nucleophilic amino acid is the defining feature of a double-displacement GT mechanism. Previous structural and functional analyses of β-Kdo GTs led to a prediction that an absolutely conserved aspartate residue (from the invariant QXXXD motif in the active site) acts as the catalytic nucleophile for these enzymes; in KpsC-N_*Ec*_, this residue is Asp160 (5, 9). The involvement of the corresponding aspartate residue in the retaining WbbB β-Kdo GT was verified experimentally (19). To further investigate the role of this residue in KpsC, WT KpsC-N_*Ec*_ and variants with Asp160 substitutions were examined for their ability to form covalent adducts with Kdo. Proteins were overproduced and purified from *E. coli* BL21 (which produces CMP-β-Kdo for the biosynthesis of LPS (20)) and examined using LC-MS before and after incubation with CMP-Kdo (which was produced *in situ*). The expected molecular weight of the WT KpsC-N_*Ec*_ enzyme construct (predicted using the ExPASy ProtParam tool) is 36,582.79 Da but, prior to incubation with CMP-Kdo, the mass spectrum for this protein shows a single species at 36,452.13 Da (Fig. 2*A*). The 130.66 Da difference is consistent with loss of the N-terminal methionine residue (Δ*m* 131.19 Da), and this explanation is supported by MS analysis after tryptic digestion of KpsC-N_*Ec*_, as well as the previously determined crystal structure of this enzyme (5). Following incubation with CMP-Kdo, the mass spectrum shows the formation of a new species with a mass increase of 219.77 Da (Fig. 2*B*), which is close to the anticipated change in mass for Kdo adduct formation (Δ*m* 220.18 Da). The native KpsC-N_*Ec*_ enzyme therefore appears capable of forming covalent Kdo adducts *in vitro*. In the absence of acceptor to drive turnover, the relatively low amounts of Asp160–Kdo adducts detected in these experiments possibly reflects the lability of this species, with water activated by the general base hydrolyzing the adduct.

To identify a more stable KpsC-N_*Ec*_-Kdo adduct, an Asp160Asn variant was analyzed. Asparagine is structurally similar to aspartate and was predicted to react with CMP-Kdo. However, as both a worse nucleophile and a worse leaving group, we rationalized that the resulting Kdo–Asn160 adduct would be significantly more recalcitrant to hydrolysis, allowing this intermediate species to accumulate. Consistent with this prediction, the D160N variant formed Kdo adducts *in vivo*, yielding two species differing by 219.66 Da in the MS profile when the protein was analyzed immediately following purification from *E. coli* BL21 (Fig. 2*C*). A similar tendency to form Kdo-adducts during protein expression was observed with WbbB (GT99) nucleophile variants (19). Incubation of the D160N variant with CMP-Kdo resulted in an apparent increase in the relative abundance of species containing the covalent Kdo adduct (Fig. 2*D*). The site of Kdo modification was mapped to a 45-residue peptide fragment obtained by tryptic-digestion (^148^TNIVLVVDQTFNNMSVTYGNAGPHEFAAMLEAAMAENPQAEIWVK^192^); this fragment contains Asn160 (underlined), consistent with this residue acting as the nucleophile. A second variant was also examined, where Asp160 was replaced with cysteine, an amino acid which is a good nucleophile, and a poor leaving group. The mass spectrum for the D160C mutant showed that it was almost completely modified with Kdo (>95%) (Fig. 2*E*), with the site of modification localized to the same 45-residue peptide identified in the D160N variant. Finally, a third KpsC-N_*Ec*_ variant was examined, where Asp160 was replaced with alanine, an amino acid lacking a nucleophilic side chain. As expected, only the nonglycosylated D160A enzyme was identified (Fig. 2*F*), even after incubation with CMP-Kdo *in vitro*. Together, these results are consistent with KpsC GTs forming Kdo adducts at the catalytically essential Asp residue of the QXXXD motif, while replacing this Asp with Asn or Cys stabilizes the adducts. In KpsC_*Ec*_, the C-terminal GT module catalyzes the formation of β-(2→7)-Kdo linkages. While catalytic Asp mutations in this module were not examined directly, investigation of the closely related *T. dismutans* β-(2→7)-Kdo GT (KpsC-C_*Td*_) revealed a high level of Kdo modification when the Asp residue of the QXXXD motif, Asp165, was substituted with Cys (Fig. S1).

**Figure 3:**
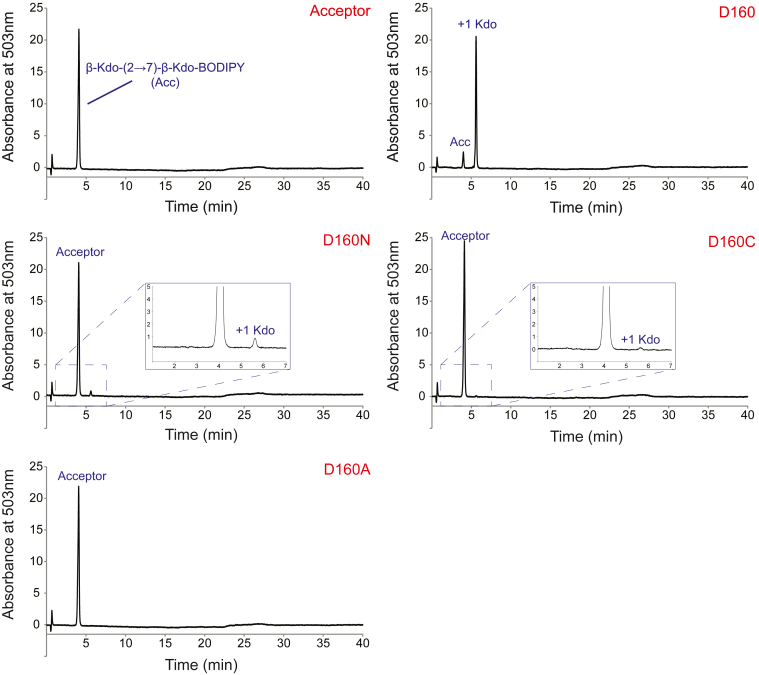
**UV-HPLC analysis of the *in vitro* β-Kdo GT activity of KpsC-N**_***Ec***_**and its site-directed mutants.** The *top left* panel shows the A_503nm_ profile for acceptor **1** in the absence of added enzyme, while the *top right* panel shows the addition of a single Kdo residue to acceptor **1** by the WT KpsC-N_*Ec*_ enzyme (labeled D160). The identity of the D160 variant is indicated in each panel in *red*. The D160N and D160C variants of KpsC-N_*Ec*_ retain minimal activity, while the D160A variant shows no detectable activity in these conditions. Reactions were incubated at 30 °C for 30 min and performed in triplicate. GT, glycosyltransferase.

To investigate whether these D160 variants remain enzymatically active, each KpsC-N_*Ec*_ variant was incubated with the CMP-Kdo reaction mixture and acceptor **1** (Kdo-β-(2→7)-Kdo-BODIPY), and reaction products were analyzed by UV-HPLC (Fig. 3). After 30 min, the WT enzyme converted most of **1** to product. In contrast, the D160N variant converted only a small amount (∼3.5% by peak integration) of the acceptor to product; the D160C converted even less (<1%) and no reaction product was detected with the D160A variant. These observations are consistent with enzyme activity being dependent on the ability of the amino acid at position 160 to form adducts and that amino acids with poorer leaving groups result in more adduct but with reduced turnover.

### Crystal structure of the KpsC-N_*Ec*_ D160N:α-Kdo adduct

**Figure 4:**
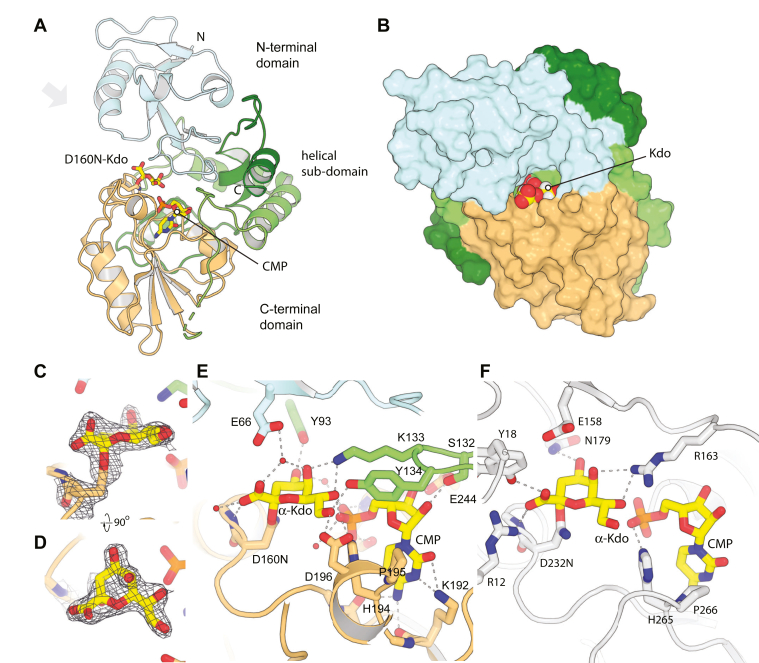
**Structure of KpsC-N D160N Kdo adduct.***A*, shows the overall structure of KpsC-N in cartoon representation, with CMP and Kdo represented in *yellow sticks*. *B*, shows a surface view, from the approximate perspective of the *gray* arrow in panel (*A*). *C* and *D*, show orthogonal views of the electron density map around the D160N–Kdo adduct, contoured at 1.0 σ. Details of interactions mediated by the D160N–Kdo adduct are highlighted in (*E*), while (*F*) presents an equivalent view of the structure of WbbB_GT99_ D232N–Kdo adduct complex.

To determine the structure of the observed KpsC-N_*Ec*_ Kdo adduct, crystals of KpsC-N_*Ec*_ D160N were grown in the presence of 1 mM CMP before being briefly soaked in a CMP-Kdo–generating reaction mixture and then immediately frozen. The resulting structure (at 1.90 Å; Fig. 4, *A* and *B* and Table 1) superimposes closely on the WT KpsC-N_*Ec*_ CMP-complex (0.28 Å rmsd, compared to PDB ID 6MGC; Fig. S2*A*), with significant shifts only occurring in surface-exposed side chains. In the resulting map, clear density was visible for the CMP nucleotide, which refined to an occupancy of 0.95. In this structure, CMP is tightly bound using an extensive network of interactions that are substantively identical to those previously reported for the KpsC-N_*Ec*_ CMP-complex (PDB ID 6MGC) (5). Additional density was associated with Asn160; this density was modeled as an α-Kdo adduct (7) and refined to a final occupancy of 0.76, with atomic displacement parameters (B-factors) similar to surrounding residues (Fig. 4, *C* and *D*). This adduct was modeled as being linked to the Oγ of Asn160. Oxygen was selected on the basis of the greater electron density for this atom, the ability of this variant to turn over in the presence of acceptor **1** (albeit much less effectively than the WT enzyme) (Figs. 4*E* and S3*A*), the precedent of an analogous linkage formed by an acetyl amide oxygen in retaining hexosaminidases (21), and the absence of a general base that might activate an asparagine Nγ nucleophile (as required in asparagine peptidyl GTs (22)). Finally, a less defined density peak stacked on Trp42 was modeled as a Kdo residue with partial occupancy (0.78), (Fig. S2*B*). This interaction with the monosaccharide appears to be nonspecific (with the Kdo probably occupying multiple conformations) and is likely driven by the high concentrations of Kdo present during soaks.

**Table 1:** Data collection and refinement statistics for KpsC-N_*Ec*_ D160N and KpsC-N_*Ec*_ D160C structures

	KpsC-N_*Ec*_ D160N	KpsC-N_*Ec*_ D160C
RCSB I. D	8FUW	8FUX
Data collection^a^		
Wavelength	0.98011	0.98011
Space group	P3_1_21	P12_1_1
Cell dimensions		
*a*, *b*, *c* (Å)	64.38, 64.38, 139.2	58.54, 80.3, 65.98
α, β, γ (°)	90, 90, 120	90, 103.04, 90
Resolution (Å)	46.4–1.90 (1.833–1.90)	48.4–1.20 (1.25–1.20)
*R*_sym_	0.08 (2.52)	0.065 (0.923)
CC_1/2_	0.998 (0.521)	0.997 (0.51)
*I*/σ*I*	12.74 (1.09)	9.0 (1.39)
Wilson B-factor	42.99	12.94
Completeness (%)	99.9 (100)	99.7 (99.8)
Unique reflections	27,092 (2660)	184,580 (18,412)
Redundancy	9.6 (9.6)	3.3 (3.2)
Refinement		
Resolution (Å)	46.4–1.9	48.4–1.20
Reflections used in refinement	27,084	184,575
Reflections used for R-free	1353	9229
*R*_work_/*R*_free_ (%)	19.05/22.57	12.38/15.68
CC_work_	0.966 (0.708)	0.978 (0.829)
CC_free_	0.986 (0.610)	0.976 (0.760)
No. of atoms		
Protein	2496	5486
Ligand/ion	89	288
Water	104	939
*B*-factors		
Protein	67.09	16.11
Ligand/ion	59.35	17.02
Water	56.90	30.09
R.m.s. deviations		
Bond lengths (Å)	0.010	0.021
Bond angles (°)	0.94	1.54
Ramachandran plot		
Favored (%)	96.8	97.8
Allowed (%)	3.2	2.1

a Each dataset was collected from a single crystal. Values in parentheses are for the highest-resolution shell.

The α-Kdo adduct is stabilized in the active site by an extensive network of hydrogen bond interactions, mediated by a series of highly conserved residues (Figs. 4*E* and S3*A*). The observed binding mode places the exocyclic O7 and O8 nearest the CMP phosphate group, while the anomeric carbon is positioned 5.4 Å away. We were unable to determine a structure for the CMP–Kdo complex because the Kdo group was mostly disordered in the D160G, D160A, and D160S CMP–Kdo soak complexes, while D160N complexes reacted too quickly to visualize the substrate and we see the adduct instead. Nevertheless, the distance between the anomeric carbon and the CMP phosphate suggests that the adduct undergoes significant structural rearrangement after transfer of Kdo from CMP. The C1 carboxylate forms a hydrogen bond with the backbone amide of the residue immediately C-terminal to Asp160 (Met161) and to two structured water molecules, one of which also hydrogen bonds to O5 and Glu66. This water molecule sits 3.7 Å from the anomeric carbon and is likely the one associated with Asp160–Kdo adduct hydrolysis. The O4 hydroxyl group hydrogen bonds with the Tyr93 phenolic hydroxyl group, as well as a water molecule that bridges to the CMP phosphate. Many of the binding determinants of the donor adduct reside in the exocyclic groups. The O7 hydroxyl hydrogen bonds with the CMP phosphate and with the Lys133 side chain Nζ (this group also hydrogen bonds with O5). In addition, the O8 hydroxyl hydrogen bonds with the side chain carboxylate of Asp196 and the side chain hydroxyl of Tyr134. The multivalent interactions the protein makes with these exocyclic groups may serve as a quality control step, helping KpsC minimize the incorporation of alternative 8 or 9 carbon CMP sugars, such as CMP-Neu5NAc in *E. coli* K1 (7).

Comparison of the Kdo adduct from KpsC-N_*Ec*_ D160N with the equivalent WbbB_GT99_ D232N (PDB ID 8CSC) (19) reveals very similar positioning and orientation of the Kdo adduct in each active site, despite none of the coordinating groups being conserved between the two enzymes (Figs. 4*F* and S3*B*). This suggests that despite the considerable divergence between KpsC (GT107) and WbbB_GT99_, this conserved presentation mode of Kdo in the active site is likely a key feature of the mechanism in both enzyme families.

### Crystal structure of KpsC-N_*Ec*_ D160C ternary complex

Crystals of a ternary complex of KpsC-N_*Ec*_ were also generated using the D160C variant. While the structure of cysteine is less similar to aspartate than asparagine, the cysteine variant converts more fully to the adduct (Fig. 2*E*) and was anticipated to be more stable on the multiweek timescale required for crystallization. The D160C variant was expressed in *E. coli* BL21 (DE3) and then crystallized in the presence of 1 mM CMP and 5 mM acceptor **2**, a β-Kdo-(2→7)-β-Kdo disaccharide (9). The structure of this complex was determined at 1.20 Å resolution; notably, this appears to be among the highest resolution structures currently determined for a GT. The resulting map revealed highly detailed density for the Cys160–Kdo adduct and for acceptor molecules in both copies of the active site present in the asymmetric unit. However, the details of ligand interactions differ between the two chains. Chain A, which contains CMP, the Kdo-adduct, and the acceptor, is the focus of the discussion below. In chain B, the acceptor is similarly bound, but CMP is absent and the Kdo-adduct is displaced from its usual site; interpretation of the data for this complex is less clear and is discussed in the SI (see Fig. S4).

**Figure 5:**
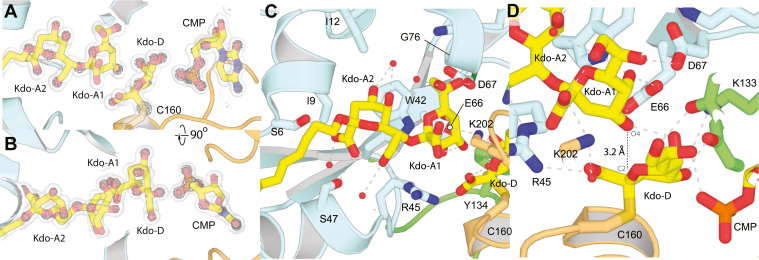
**Structure of KpsC D160C ternary complex.***A* and *B*, show orthogonal views of the electron density map around the ligands in the D160C–Kdo ternary complex. The transparent surface is contoured at 1 σ, and the mesh is contoured at 4 σ. Note that most heteroatoms have clear, distinct peaks at 4 σ. Panel (*C*) shows details of interactions mediated by the D160C–Kdo acceptor disaccharide and (*D*) highlights the organization of the acceptor Kdo-A1 group and the Kdo–C160 adduct.

Overall, the KpsC–N_*Ec*_–D160C ternary complex superimposes closely on the KpsC–N_*Ec*_ CMP complex (PDB ID 6MGC), but the N-terminal domain (residues 2–70) undergoes an approximately 8° rigid body rotation, which narrows the active site cleft by roughly 1.5 Å (Fig. S2*C*). The CMP nucleotide is bound with an occupancy 0.97 in chain A. The α-Kdo adduct (occupancy 1.00) is positioned in the active site in a similar orientation to the D160N structure (Fig. 5, *A* and *B*). Cys160 Cα is shifted roughly 1 Å into the catalytic pocket to compensate for its shorter side chain (compared to aspartate or asparagine), and the anomeric carbon (C2) is shifted 0.5 Å towards Asp/Asn/Cys160. Overall, the positioning of Kdo is close to that described for the D160N structure, and most interactions are equivalent in the two structures. One noticeable difference is that the C1 carboxylate rotates ∼90° and hydrogen bonds with the guanidinium group of Arg45. This residue partially blocks the acceptor binding site in the KpsC-N_*Ec*_-D160N Kdo adduct structure and is shifted into the active site by both the rotation of the N-terminal domain and reorganization of the Arg45 side chain (Figs. 5*C* and S3*C*).

In our refined ternary structure, acceptor **2** bound with full occupancy in the active sites of both chains, with the aglycone ordered by interactions with an exposed hydrophobic patch on a lattice-related protein chain (Fig. S2*D*). In the following analysis, we designate the Cys-linked donor Kdo as Kdo-D, the nonreducing terminal acceptor Kdo (that attacks the donor) as Kdo-A1, and the additional ordered acceptor Kdo as Kdo-A2.

The acceptor disaccharide interacts almost exclusively with residues from the N-terminal α/β domain (Fig. 5, *C* and *D*). The Kdo-A2 pocket is relatively nonpolar, with the Kdo-A2 C4, C5, and C6 stacking on the indole ring of Trp42, C3 stacking on Gly8 and Ile9. Hydrogen bonds are observed between the C1 carboxylate and the hydroxyl groups of Ser6 and Ser47; the O4 carboxylate also hydrogen bonds to O8 within Kdo-A2, which in turn hydrogen bonds with Arg45. Finally, a pair of ordered water molecules bridge O8 and Ser47N and O4 and O5. In contrast with the Kdo-A2–binding site, the Kdo-A1–binding site is polar, with the monosaccharide stabilized by multiple hydrogen bonds. The C1 carboxylate group of Kdo-A1 is hydrogen bonded to Lys202 (the only directly interacting residue from outside the N-terminal α/β domain), as well as to Kdo-A1 O8. The presence of an electropositive amino acid (Lys/Arg) at this position is highly conserved among KpsC homologs (5), suggesting that this interaction is important for positioning and stabilizing the acceptor. The exocyclic O7 and O8 atoms of Kdo-A1 pack against Gly76, with O7 also forming a hydrogen bond with Asp67. Kdo-A1 O5 hydrogen bonds with the indole N–H of Trp42, as well as the Glu66 carboxylate; Trp42, Glu66, and Asp67 are all absolutely conserved among both modules of every KpsC homolog (5). Glu66 also hydrogen bonds with the O4 hydroxyl group and with the Kdo-D O5 hydroxyl. The O4 hydroxyl group of Kdo-A1 is located 3.2 Å away from the anomeric carbon of α-Kdo-D and positioned exactly in line with the C2-Cys160 S_γ_ bond; it is therefore well-positioned for nucleophilic attack on the donor (Figs. 5*D* and S3*D*). The ideal hydrogen bond with Glu66 (2.7 Å, with donor, acceptor, and inferred hydrogen directly in line) should allow this group to act as an effective nucleophile. The organization of this ternary complex is consistent with Kdo-A1 O4, performing a nucleophilic attack on the anomeric carbon of the α-Kdo-D adduct species. An S_*N*_2 reaction arising from this complex would produce a new β-(2→4) linkage between Kdo-A1 and Kdo-D, as observed in the reaction product (5). This suggests that this complex is a productive one, and that KpsC-N, like WbbB_GT99_, uses a double displacement mechanism.

### The N-terminal α/β domains of KpsC GTs dictate both acceptor and linkage specificity

**Figure 6:**
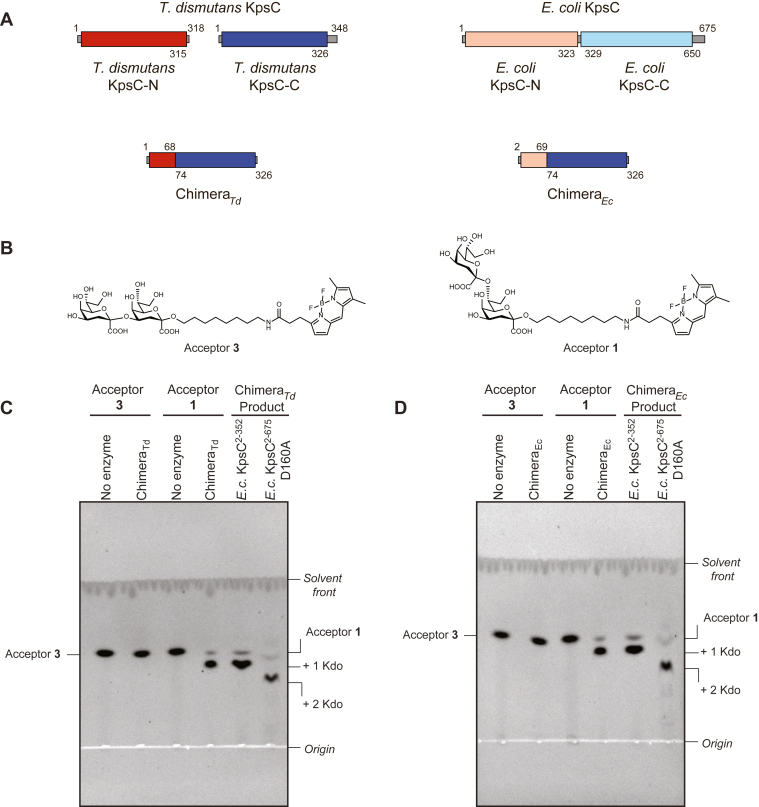
**Functional characterization of KpsC chimeras.***A*, generation of KpsC chimeras from characterized KpsC-N and KpsC-C enzymes from *Thermosulfurimonas dismutans* and *Escherichia coli*. *B*, enzyme acceptors containing β-Kdo disaccharides with either (2→4)- (acceptor **3**) or (2→7)-linkages (acceptor **1**). *C*, TLC analysis of the reaction product synthesized by *T. dismutans* KpsC chimera (Chimera_*Td*_) and *E. coli* KpsC enzymes with established specificity. *D*, equivalent TLC analysis of the *E. coli* KpsC chimera (Chimera_*Ec*_).

In our previous study, the structure of apo-KpsC-C_*Td*_ was found to differ significantly from KpsC-N_*Ec*_ in the architecture of the N-terminal α/β domain, but both proteins shared a common core of conserved residues in what is now identified (above) as the acceptor-binding site in KpsC-N_*Ec*_. The acceptor-bound structure of KpsC-N_*Ec*_ clearly shows that almost all acceptor-coordinating interactions are mediated by the N-terminal α/β domain (residues 2–70). This suggests (possibly subtle) structural differences in the N-terminal domains of the KpsC-N and KpsC-C GT modules determine acceptor specificities and binding geometries, which in turn dictate new linkages with distinct hydroxyl groups. The importance of the N-terminal domains in dictating acceptor specificity of GT-B fold GTs has been reported previously with chimeric enzymes from glycopeptide biosynthesis (23). To investigate the consequences for KpsC, recombinant chimeric enzyme constructs were generated by exchanging the N-terminal α/β domains between KpsC-N and KpsC-C GTs and then analyzing the corresponding acceptor and linkage specificity. Two functional chimeras were generated by combining the N-terminal α/β domains domain from either KpsC-N_*Td*_ or KpsC-N_*Ec*_ with KpsC-C_*Td*_ residues 74–326 (Fig. 6*A*). Chimera_*Td*_ contains residues 1–68 of KpsC-N_*Td*_, whereas Chimera_*Ec*_ possesses KpsC-N_*Ec*_ residues 2–69. The activities of these enzymes were then characterized. Unfortunately, attempts to generate active chimeric enzymes containing the N-terminal α/β domain from the KpsC-C GT module were unsuccessful. This presumably reflects an inappropriate selection of the fusion point and loss of proper folding and/or important interdomain interactions.

**Figure 7:**
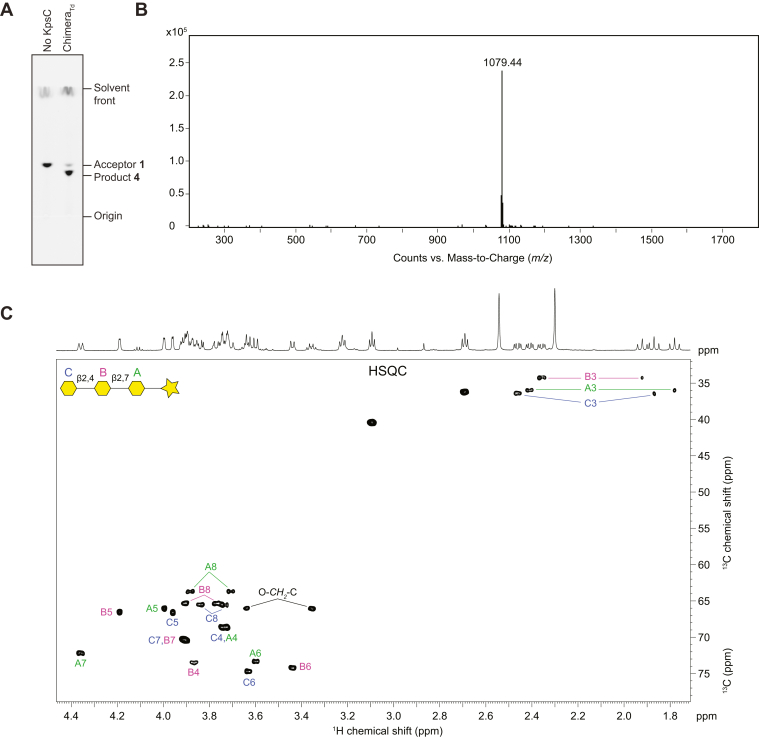
**Structural characterization of product 4 synthesized by Chimera**_***Td***_**.** Panels (*A*) and (*B*) show TLC separation and the charge-deconvoluted ESI mass-spectrum of reaction product **4**, respectively. The calculated monoisotopic mass of **4** is 1079.43. Panel (*C*) shows a selected region from the ^1^H,^13^C HSQC spectrum of product **4**. Kdo residues are designated by letters, and the signals were assigned by comparison to previously characterized synthetic Kdo trisaccharide products (5, 9). Downfield shifts of the substituted carbon atoms in Kdo residues (due to a positive α-glycosylation effect) defined the positions of substitution.

Enzyme reactions were performed using the KpsC chimeras with synthetic acceptors **1** and **3**. Acceptor **1** contains a β-Kdo-(2→7)-β-Kdo disaccharide, whereas acceptor **3** contains a β-Kdo-(2→4)-β-Kdo disaccharide; each is linked to a BODIPY tag (Fig. 6*B*). Chimera_*Td*_ and Chimera_*Ec*_ both lacked activity with acceptor **3** but catalyzed addition of a single Kdo residue to acceptor **1**, which was evident in TLC and ESI MS analyses (Figs 6, *C* and *D* and 7, *A* and *B*). To confirm the linkages catalyzed by these chimeric enzymes, the reaction products were isolated and secondary reactions were performed. Secondary reactions exploited the recombinant *E. coli* KpsC^2-352^ and KpsC^2-675^ D160A enzymes, whose activities were established previously (9). *E. coli* KpsC^2-352^ contains KpsC-N and only recognizes a β-Kdo-(2→7)-β-Kdo acceptor. This enzyme was unable to extend either of the chimera reaction products. In contrast, the D160A variant of *E. coli* KpsC^2-675^ possesses an inactivated KpsC-N but retains a functional KpsC-C and so only recognizes β-Kdo-(2→4)-β-Kdo acceptors. This enzyme catalyzed the addition of a single Kdo residue to both chimera reaction products, indicating Chimera_*Td*_ and Chimera_*Ec*_ are functionally identical and catalyze addition of a β-(2→4)-Kdo. To confirm the identity of KpsC chimera product, we performed a scaled-up reaction using Chimera_*Td*_ and acceptor **1**, which was monitored by TLC (Fig. 7*A*). The obtained product **4** was then purified and analyzed by ESI MS and ^1^H and ^1^H,^13^C heteronuclear single quantum coherence (HSQC) NMR spectroscopy (Fig. 7, *B* and *C*). ^1^H and ^13^C NMR chemical shifts for the carbohydrate moiety of **4** were nearly identical to those in a previously characterized trisaccharide product containing β-Kdo-(2→4)-β-Kdo-(2→7)-β-Kdo (Table S1) (11).

These experiments confirmed Chimera_*Td*_ is functionally equivalent to native KpsC-N enzymes, which add β-(2→4)-Kdo residues (5, 9). Furthermore, these results established that residues within the N-terminal α/β domain of KpsC (residues 1–70), which forms an acceptor-binding domain, dictate both acceptor preference and the specific linkage catalyzed by the enzyme.

### AlphaFold model of full-length KpsC

**Figure 8:**
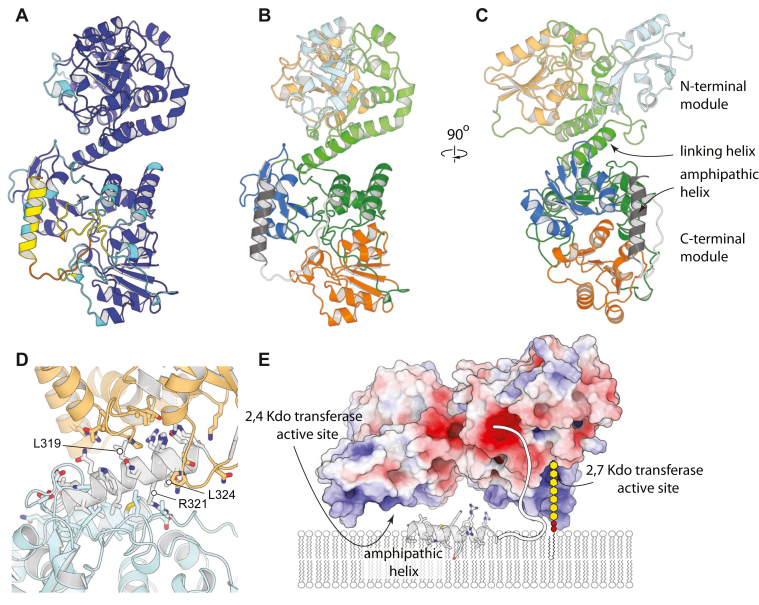
**AlphaFold model of full-length *E. coli* KpsC.***A*, KpsC colored by pLDDT; (*blue*: pLDDT > 90, *cyan*: pLDDT > 70, *yellow*: pLDDT > 50, *orange*: pLDDT <50). *B* and *C*, orthogonal views of KpsC, colored by module and domains within each module. The N-terminal α/β domain in *blue*, the helical inserts in *green*, and the C-terminal α/β domain in *orange*. The C-terminal β2,7-Kdo transferase is shown in darker shades than the N-terminal β-(2→4)-Kdo module. The C-terminal amphipathic helix is shown in *dark gray*, and the linker joining it to the β-(2→7)-Kdo module is shown in *white*. Note that the amphipathic C-terminal helix is attached by a disordered tether and likely does not pack on the rest of the structure. *D*, details of the interaction between domains. N-terminal domain is in *cyan*, C-terminal domain in *orange*, and the linking helix in *white*. *E*, model of KpsC interactions with the membrane. A cartoon representation of the substrate models how the substrate might reach the active sites.

To date, dual module constructs of KpsC have proven recalcitrant to crystallization. To gain a better understanding of the structure of the full-length enzyme, we retrieved the model of *E. coli* KpsC (AF-AOAOH2Z2W) from the AlphaFold database (24). This model is generally well predicted, with pLDDT scores >90, except for the very C-terminus, which appears to be partially disordered (Fig. 8, *A*–*C*). In this model, the N-terminal module superimposes closely on the *E. coli* KpsC-N experimental structures (*e.g.* 0.30 Å rmsd vs 6MGC, the CMP costructure). The C-terminal module superimposes considerably less well (3.54 Å rmsd over 271 residues) on the structure of the monofunctional β-(2→7)-Kdo transferase from *T. dismutans* (PDB ID 6MGD), with the differences possibly representing either structural divergence between homologs or differences in structural state (the AlphaFold model seems to represent a more ordered, turn-over competent state). The two GT modules appear to be structurally linked but only interact by packing on opposite faces of a shared five-turn α-helix (Fig. 8*D*). Residues 310–318 of this linking helix pack on the KpsC-N module and bury 869.7 Å^2^, including a hydrophobic core; Arg321 also reaches across and forms a single salt bridge with Asp130. Residues 319–327 interact predominantly with the KpsC-C module, burying 621.1 Å^2^; most of the interactions are through hydrogen bonds, although Leu319 and Leu324 contribute some nonpolar interactions. The interactions between the linking helix and the KpsC-C module seem relatively weak, and indeed, PISA analysis indicates that this interface yields only 1.4 kcal/mol of hydrophobic binding energy. Considered with the disorder of the C-terminal region of this helix in structures of the KpsC-N domain, the model suggests that the two GT modules might retain some ability to reorient relative to one another by partially unwinding this helix. This arrangement contrasts with the only other structurally characterized dual-module GTs, *E. coli* chondroitin polymerase (25) and *Klebsiella pneumoniae* WbbM (26), where the two GT modules interact through extensive intermodule interfaces. However, like these two other proteins, KpsC organizes both catalytic sites to lie on the same face of the protein. Because the substrates of these GTs are membrane anchored, having both active sites on a single face likely confers a kinetic advantage.

At the C-terminus of KpsC, residues 657–675 form a five-turn, amphipathic α-helix comprised of only basic or nonpolar residues. This helix likely functions as a membrane anchor (see below). Residues 644–656 form a linker (pLDDT < 50) connecting this helix to the C-terminal KpsC GT module. Interestingly, the linker is also composed almost exclusively of basic and nonpolar residues, suggesting that it might also participate in membrane association. Because this linker emerges at the periphery of the C-terminal active site, it is conceivable that it may preferentially position the protein so that the active sites face the membrane. Of note, the region surrounding both active sites is electropositive, which might help both drive association with the membrane periphery and help recruit its polyanionic acceptor substrate. A potential model of KpsC’s interactions with the membrane is shown in Figure 8*E*.

### The C-terminal amphipathic helix in KpsC-C enzymes influence *in vitro* product distribution

**Figure 9:**
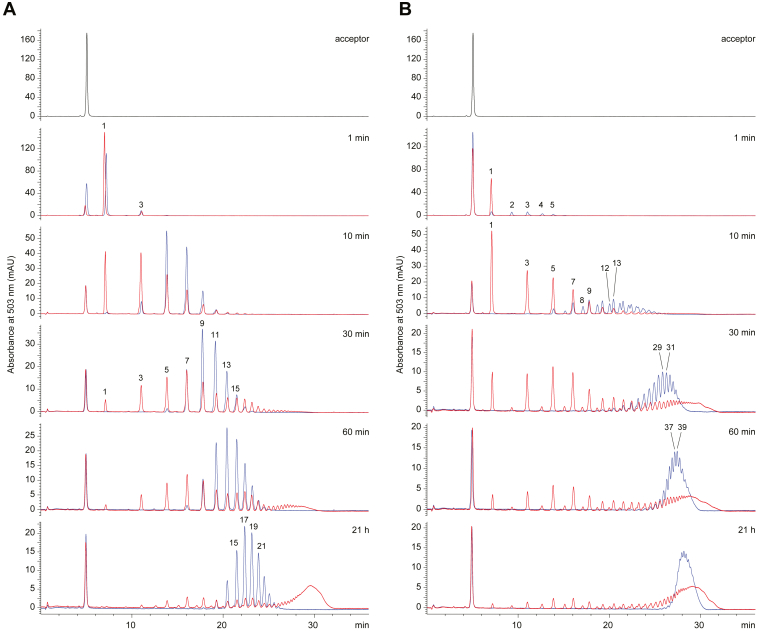
**The C-terminal amphipathic helix of dual-module KpsC enzymes influences the size distribution of *in vitro* oligo-Kdo product.** The panels show the HPLC profiles of reaction products from a time course experiment with *Escherichia coli (A)* and *Actinobacillus pleuropneumoniae*. *B*, KpsC enzymes incubated with CMP-Kdo and acceptor **1**. The reactions were performed using full-length proteins (*blue*) or a truncated derivatives (KpsC_Δhelix_), which contain both GT modules but have the predicted C-terminal amphipathic helices removed (*red*). Reactions were incubated at 30 °C and performed in triplicate with similar results. Numbers indicate the number of Kdo resides transferred to acceptor **1**. The small peak of unreacted acceptor that remained unchanged over the time course is due to contaminating α-Kdo-(2→7)-β-Kdo-BODIPY, which is not a substrate for KpsC. GT, glycosyltransferase.

All currently identified KpsC enzymes possess a C-terminal amphipathic helix, which is predicted to drive membrane association as described above. Removal of this amphipathic helix results in soluble protein that retains both β-(2→4)- and β-(2→7)-Kdo transferase activities, but the potential influence of this region on overall activity has not been examined. To address this question and confirm that any involvement was not confined to a specific homolog, the amphipathic helix was removed from two different KpsC enzymes obtained from *E. coli* K1 and a livestock pathogen, *A. pleuropneumoniae.* Full-length KpsC and the truncated constructs (KpsC_Δhelix_; *E. coli* KpsC^2-650^ and *A. pleuropneumoniae* KpsC^2-670^) were incubated with CMP-Kdo and acceptor **1**, and the formation of products was monitored over a time course from 0 − 21 h by HPLC (Fig. 9). In general, *A. pleuropneumoniae* KpsC directs a higher degree of *in vitro* polymerization than its *E. coli* counterpart, with >40 Kdo residues added overnight, compared to an average of 17 − 19 Kdo for the *E. coli* enzyme. The products generated by *E. coli* KpsC possess β-(2→4)-Kdo at the nonreducing terminus (established by an odd number of residues added to the known acceptor structure), in accordance with previous studies (5, 9). While *A. pleuropneumoniae* KpsC retains the preference for β-(2→4)-Kdo at the nonreducing terminus of products, some products were also apparent with terminal β-(2→7)-Kdo, identified by even numbers of residues added to the acceptor. The range of reaction products suggest the *A. pleuropneumoniae* KpsC enzyme contains a KpsC-C GT module with increased efficiency, relative to its *E. coli* counterpart.

Both *E. coli* and *A. pleuropneumoniae* KpsC produce relatively narrow product distribution at every time point, characteristic of a distributive mechanism. Interestingly, removing the C-terminal amphipathic helix resulted in transition to a processive product profile for both enzymes. This profile is characterized by simultaneous presence of short oligo-Kdo products (1, 3, 5 Kdo additions) and substantially longer polymers (over 40 Kdo additions at 21 h). Therefore, it appears that removing the C-terminal amphipathic helix increases the affinity of KpsC for longer-chain products *in vitro*.

## Discussion

Bacterial cell surface glycostructures are important virulence determinants and their exposure on the cell surface has made them attractive as vaccine targets. However, vaccine development is challenging because of the enormous structural and antigenic diversity of these polysaccharides among different species or serotypes within a species, as well as examples of host mimicry. Small molecule inhibitors of capsule biosynthesis offer a valid alternative (13), but this approach depends on identifying targets spread across serotypes and (preferably) species. KpsC is a potential therapeutic target because it is an essential component for assembling capsular structures in a diverse range of bacteria and homologs are absent from eukaryotes. However, understanding the mode of action of possible KpsC inhibitors and pursuing rational design of inhibitors are advanced by detailed understanding of the enzyme’s mechanism and biochemical characteristics. In this study, we present evidence that in KpsC (specifically KpsC-N), the donor Kdo is transferred from CMP-β-Kdo to Asp160, forming an Asp160-α-Kdo adduct, prior to transfer to the poly-Kdo acceptor. Therefore, like the distantly related β-Kdo transferase WbbB (19), KpsC operates *via* a double-displacement mechanism, rather than the S_*N*_i mechanism otherwise generally observed in retaining GTs. The evolutionary origin of these enzymes is unclear. As a group, β-Kdo GTs represent a radical departure from typical GT-B enzymes, so a search of the PDB with DALI returns no additional proteins with global resemblance. They share an HP motif with some CMP-sialic acid–dependent inverting GTs, but we have not been able to identify any other potentially conserved motifs shared with other GT-B enzymes. Known retaining β-Kdo GTs show high sequence divergence resulting in a phylogenetic tree with long terminal branches, precluding deeper phylogenetic insight (5, 27). Indeed, prior to their initial biochemical characterization (7, 9), they were not annotated as GTs based on sequence alone.

**Figure 10:**
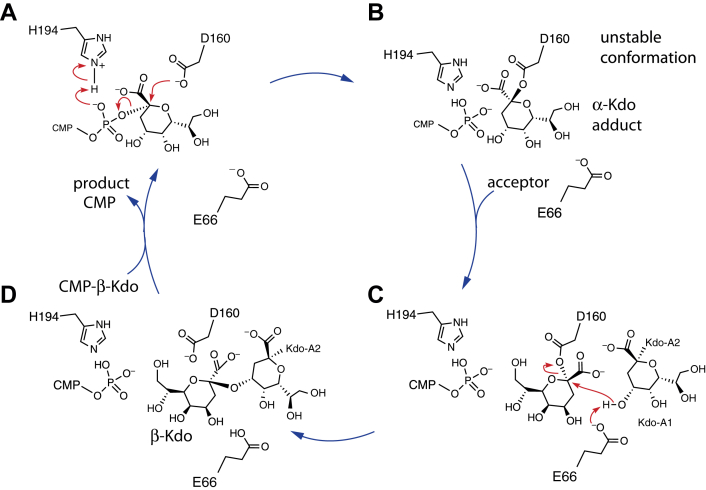
**Proposed mechanism of KpsC-N**. *A*, the anomeric carbon of the donor Kdo is attacked by Asp160; His94 acts as a general acid, promoting departure of the phosphate group. *B*, the immediate product of the reaction is an unstably bound adduct. *C*, the adduct rearranges into a more stable configuration. After binding the acceptor, O4 of Kdo-A1 is activated by Glu66 and attacks the anomeric carbon of the Kdo adduct. *D*, the resulting product can then be released, allowing binding of the next CMP-Kdo donor.

To summarize the proposed mechanism for KpsC-N, the reaction is initiated by Asp160 performing a nucleophilic attack on the anomeric carbon of the Kdo group of CMP-β-Kdo, with His94 acting as a general acid to protonate the phosphate of the CMP leaving group (Fig. 10*A*). The resulting Asp160-β-Kdo adduct then repositions itself in the active site (Fig. 10*B*), occupying a second site with the anomeric carbon rotated 5.4 Å away from the CMP phosphate. This state is then preorganized for binding of the disaccharide Kdo acceptor, with the acceptor interacting primarily with the N-terminal α/β domain. The O4 group of the nonreducing terminal Kdo of the acceptor hydrogen bonds with Glu66, which acts as a general base (Fig. 10*C*). O4 then acts as a nucleophile, attacking the Asp160-β-Kdo group, resulting in the addition of a new β-(2→4)-linked Kdo group to the acceptor (Fig. 10*D*). The KpsC reactive site is therefore proposed to be composed of two distinct half sites, each with its own catalytic machinery for carrying out the two distinct half reactions. Of note, this mechanism opens up new potential strategies for targeting KpsC. In particular, the presence of a strong nucleophile in the enzyme, and the use of a covalent intermediate, suggest it may be susceptible to inhibition by electrophilic substrate mimics to form dead-end covalent adducts. Because the human genome encodes no apparent mechanistically similar enzymes, it may be possible to develop inhibitors with very narrow substrate specificity.

While most of the data presented here concern the KpsC-N GT module, the key residues that surround the CMP-β-Kdo–binding site catalyze the first half reaction and coordinate the α-Kdo intermediate in KpsC-N_*Ec*_ and are essentially identical in the KpsC-C_*Td*_ GT module. This includes the HP and QXXXD motifs and Tyr97, Lys137, and Tyr138 in the helical subdomain (the equivalents of Tyr93, Lys133, and Tyr134 in KpsC-N_*Ec*_, respectively). This, along with the demonstration that KpsC-C can form a C165-Kdo adduct, supports a model where KpsC-C_*Td*_ also utilizes a similar double-displacement mechanism, with the structural states leading up to acceptor binding closely resembling those formed by the KpsC-N module.

The data presented here leads to the conclusion that the N-terminal domain of each GT module alone controls acceptor binding and linkage specificity. This is consistent with the near absolute conservation of key motifs in the donor-binding C-terminal α/β domain, as well as the observation that the acceptor in the KpsC-N module interacts almost exclusively with the much more strongly divergent N-terminal domain. However, the most compelling evidence for this conclusion is provided by the presented biochemical experiments showing that the linkage specificities (β-(2→4)-Kdo and β-(2→7)-Kdo) in chimeric enzymes are dictated solely by the source of the N-terminal domain. This concept has been exploited previously in the creation of chimeric enzymes with new specificities using GT-B fold GTs from glycopeptide biosynthesis (23). Our data supports the validity of the chimera approach in engineering enzymes with new activities to synthesize defined glycostructures for fundamental and applied research. The precise acceptor recognition mode of KpsC-C_*Td*_ has not been resolved. The need for the acceptor to both be activated as a nucleophile by Glu70 and to position its O7 hydroxyl group adjacent to the Kdo adduct anomeric carbon constrains possible acceptor models. However, it is not clear how the next acceptor Kdo residue would be recognized or why (2→4) linked substrates might be excluded. Unfortunately, while we were able to grow crystals of the KpsC-C_*Td*_ D165C variant with a covalent Kdo-adduct in the presence of a β-Kdo-(2→4)-β-Kdo-(2→7)-β-Kdo trisaccharide acceptor, these crystals did not yield usable diffraction data.

Removal of the C-terminal amphipathic helix from KpsC results in an enzyme that is soluble and more amenable for applications screening and inhibitor development. This change also alters the product profile. The AlphaFold model suggests that the changes in product length distribution are unlikely to be due to perturbation of the active site by the C-terminal amphipathic helix, and the changes may simply reflect altered interactions between enzyme and the nascent chains. The natural acceptor is a membrane-embedded glycolipid, rather than a soluble synthetic conjugate, and the activity of KpsC is also constrained by the intervention of enzymes that add the serotype-specific glycan. As a result, while the effect of the helix deletion is interesting, it is not possible to directly translate the *in vitro* product profile to the mechanism by which product length is regulated within the cell. Nevertheless, understanding the baseline product profile is important in developing and evaluating small-molecule inhibitors.

Collectively, the data reported here offer substantial new insight into the mechanism of action and determination of specificity of β-Kdo GTs and identify concepts that may be relevant for other GTs. These studies provide an essential foundation for the exploitation of KpsC as a therapeutic target.

## Experimental procedures

### Bacterial strains and growth conditions

Unless otherwise indicated, bacteria were grown at 37 °C in LB medium (Invitrogen) containing (where appropriate) one or more of the following supplements: kanamycin (50 μg/ml) and IPTG (0.3 mM). *E. coli* DH5α (φ80d *deoR lacZΔM15 endA1 recA1 hsdR17* (r_x_^−^ m_x_^+^) *supE44 thi-1 gyrA96 relA1* Δ(*lacZYA*-*argF*)*U169* F^−^) (Invitrogen) was used as a host for cloning. Protein expression was performed in *E. coli* BL21 (DE3) (F ^−^ *ompT gal dcm hsdS*_*B*_ (r_B_^−^ m_B_^−^) λ(DE3)) (Novagen).

### General DNA methods

The plasmids used in this study are described in Table S2. They were maintained in *E. coli* DH5α, grown at 37 °C in LB medium containing 50 μg/ml kanamycin. KOD or Phusion DNA polymerase was used to PCR amplify DNA fragments for cloning and site-directed mutagenesis. For cloning, oligonucleotide primers (Table S3) incorporated restriction sites and sequences encoding epitope tags. GeneJET PCR Purification (Thermo Fisher Scientific) kits were used to purify PCR fragments, and GeneJET Plasmid Purification (Thermo Fisher) kits were used to purify plasmids. Restriction endonucleases (Invitrogen and New England Biolabs) were used as recommended by the manufacturers. Site-directed mutagenesis was performed using the Quik-change site-directed mutagenesis strategy (Agilent) according to the manufacturer’s recommendations. The *Actinobacillus pleuropnumoniae kpsC* gene was codon-optimized for expression in *E. coli* and synthesized by GeneArt Gene Synthesis service based on the sequence in Accession number ABN74779. The *E. coli* KpsC sequence was taken from Accession Number KIE82100. Recombinant plasmids containing genes encoding chimeric KpsC proteins were constructed by cloning PCR fragments into pET28a(+) *via* Gibson Assembly, according to manufacturer’s instructions. The sequences of all plasmid constructs were verified at the Genomics Facility in the University of Guelph Advanced Analysis Centre.

### Protein overproduction and purification

Overnight cultures of *E. coli* BL21 (DE3) transformants containing the appropriate plasmids (Table S2) were used to inoculate 0.25 to 1 L of LB medium, supplemented with 50 μg/ml kanamycin, at a 1:100 dilution. Cultures were grown at 37 °C until an A_600nm_ of ∼0.6 was achieved. Expression of the recombinant gene was induced by adding IPTG to a final concentration of 0.3 mM, and the cells were left to grow for 2.5 to 3 h at 37 °C. In all cases, cells were harvested by centrifugation at 5000*g* for 10 min and stored at −80 °C until needed. The cell pellet was resuspended in 25 ml of buffer A (20 mM Tris–HCl, 500 mM NaCl, 10% glycerol, pH 8.0) supplemented with Complete mini EDTA-free protease inhibitors (Roche Applied Science). Cells were lysed by ultrasonication for a total of 3.5 min with 30% amplitude in pulses (10 s on/15 s off). The suspension was cleared *via* successive centrifugation steps at 4000*g* for 10 min, 12,000*g* for 20 min, and 100,000*g* for 1 h at 4 °C. Proteins were purified directly from the residual supernatant from the final centrifugation step using 2 ml of nickel-nitrilotriacetic acid-agarose resin columns (Qiagen). The column was conditioned initially with 10 column volumes of buffer A before sample was applied. The column was then washed with 10 column volumes each of buffer B (buffer A containing 30 mM imidazole), before elution with 10 column volumes of buffer C (buffer A containing 500 mM imidazole); eluent was collected in 1-ml fractions. Protein purity was assessed using SDS-PAGE with SimplyBlue SafeStain (Life Technologies). Protein was buffer exchanged using a PD-10 desalting column and concentrated using a Vivaspin centrifugal concentrator. Proteins used for *in vitro* biochemical experiments were buffer exchanged into buffer A, whereas proteins used in x-ray crystallography experiments were buffer exchanged as described below. Protein concentrations were estimated from A_280nm_ values using theoretical extinction coefficients predicted by the ExPASy ProtParam tool (https://web.expasy.org/protparam/). For long-term storage, proteins were placed in buffer A and stored at −80 °C.

### Protein LC-MS analysis of KpsC enzymes

KpsC proteins and their variants were analyzed using LC-MS on an Agilent 1260 HPLC Liquid Chromatograph interfaced with an Agilent UHD 6530 Q-Tof Mass Spectrometer at the Mass Spectrometry Facility of the Advanced Analysis Centre, University of Guelph. A Zorbax 300SB-C18 column (1 × 50 mm, 3.5 μm, Agilent) was used for separation of protein from detergents with the following solvents: water with 0.1% formic acid for A and acetonitrile with 0.1 % formic acid for B. The mobile phase gradient was as follows: initial conditions, 10% B for 5 min, increasing to 85% B in 7 min and then to 95% B in 0.10 min followed by column wash at 95% B and 5 min re-equilibration. The first 2 min and last 5 min of gradient were sent to waste. The flow rate was maintained at 0.3 ml/min. The mass spectrometer electrospray capillary voltage was maintained at 5.0 kV and the drying gas temperature at 350° C with a flow rate of 13 l/min. Nebulizer pressure was 40 p.s.i., and the fragmentor was set to 300 V. Nitrogen (purity > 98%) was used as nebulizing, drying, and collision gas. The mass-to-charge ratio was scanned across the *m/z* range of 300 to 3200 *m/z* in 4 GHz (extended dynamic range) positive-ion mode. The instrument was externally calibrated with the ESI TuneMix (Agilent). The sample injection volume was 2 μl. Data analysis was performed using the MassHunter Qualitative Analysis Version B.06.00 (Agilent; https://www.agilent.com/Library/usermanuals/Public/G3336-90024_Qual_Familiarization_GCMS.pdf) software. Deconvolution of the *m*/*z* spectrum was achieved using the pMod algorithm within the BioConfirm software (Agilent; https://www.agilent.com/en/product/software-informatics/mass-spectrometry-software/data-analysis/bioconfirm-software).

For tryptic digestion of KpsC-N_*Ec*_ enzymes, modified trypsin was used where autolysis has been blocked and chymotryptic activity inhibited (Fisher Sequencing Grade Modified trypsin V5111). Twenty micrograms of trypsin were dissolved in 20 mM acetic acid to a final concentration of 0.2 μg/μl. When ready for digestion, 50 mM ammonium bicarbonate was added such that the enzyme working solution was 0.01 μg of enzyme. Trypsin was added in a 1:10 ratio of protease to protein and incubated at 37 °C overnight. Mass spectrometry was as above, except that a C18 column (Agilent 32 AdvanceBio Peptide Map, 100 mm × 2.1 mm, 2.7 μm) was used for chromatographic separation (at 0.2 ml/min), and the mobile phase gradient was as follows: initial conditions, 2% B, increasing to 45% B in 40 min and then to 55% B in 10 more minutes followed by column wash at 95% B and 10 min re-equilibration. Three precursor ions per cycle were selected for fragmentation. Raw data files were loaded directly into PEAKS 8.5 software (Bioinformatics Solutions Inc; https://www.bioinfor.com/peaks-85-release/), where the data was refined and subjected to *de novo* sequencing and database searching. The following modifications were considered within the search parameters: deamidation of asparagine and glutamine (+0.98 Da), oxidation of methionine (+15.99 Da), and Kdo glycosylation (+220.06 Da). The tolerance values used were 15 ppm for parent ions and 0.05 Da for fragment ions. Data was searched against the WT KpsC-N_*Ec*_ and variant sequences.

### Synthesis of acceptors 1 to 3

Acceptor **1** was prepared as previously described (14). The chemical synthesis of **2** and enzymatic synthesis of **3** are described in the Supporting Information text and Fig. S5.

### UV-HPLC analysis of KpsC-N_*Ec*_ variant activities

*In vitro* reactions were performed in 40 μl volumes and contained 50 mM Hepes (pH 8.0), 10 mM MgCl_2_, 0.5 mM Kdo, 1.25 mM CTP, 5 μM acceptor **1**, 4 μg of KdsB, and 4 μg of KpsC-N_*Ec*_ or its variants (D160N, D160C, or D160A). The reaction mixtures were incubated at 30 °C for 30 min. Ten microliters of aliquots were removed and stopped by adding equal amounts of cold acetonitrile, followed by removal of precipitated protein by centrifugation at 13,000*g* for 5 min. HPLC analysis was performed on an Agilent 1260 Infinity II LC system equipped with a 1260 Infinity II Variable Wavelength Detector. A DNAPac PA-100 column (4 × 50 mm) was used for chromatographic separation with the following solvents: water (A), acetonitrile (B), and 2 M ammonium acetate (C). The mobile phase gradient was as follows: 20% B was kept constant throughout the run; initial conditions were 0% C; increasing to 50% C in 15 min; hold 50% C for 5 min; decreasing to 0% C in 5 min; 15 min of re-equilibration. The flow rate was maintained at 0.5 ml/min, and the column temperature was set to 40 °C. The elution profile was monitored by UV detection at 503 nm and the injection volume was 8 μl. These analyses were performed in triplicate.

### Determination of the structure of *E. coli* KpsC^2-323^ D160N and D160C by x-ray crystallography

All crystallization experiments were performed using a sitting-drop configuration at room temperature. Protein was buffer exchanged into a 20 mM Tris–HCl, 150 mM NaCl, 1 mM CMP, pH 7.5 solution and concentrated. *E. coli* KpsC^2-323^ D160N was mixed in a 1:1 ratio (2 μl drop volume) with the well solution and equilibrated against ∼100 μl of the same well solution. *E. coli* KpsC^2-323^ D160N (at 15 mg/ml) was crystallized using a well solution containing 0.2 M lithium sulfate, 29 % (v/v) PEG 3350, 0.1 M Bis-Tris, pH 5.5, and 2 mM strontium acetate. To generate CMP-Kdo, a reaction containing 50 mM Hepes, 5 mM MgCl_2_, 4 mM Kdo, 10 mM CTP, and 4.5 μg of KdsB from *E. coli* was incubated for 2.5 min before soaking crystals with the reaction mixture for 8 min at room temperature in a well, where the PEG 3350 concentration had been increased to 33 %, prior to cryoprotection. *E. coli* KpsC^2-323^ D160C was mixed with 5 mM acceptor **2** and incubated at room temperature for 30 min prior to crystallization. *E. coli* KpsC^2-323^ D160C (at 10 mg/ml) was crystallized using a well solution containing 25 % (v/v) PEG 3350 and 0.1 M Bis-Tris, pH 5.5; protein was mixed in a 1:1 ratio (0.3 μl drop volume) with the well solution and equilibrated against ∼40 μl of the same solution. All crystals were immersed in paratone N oil for cryoprotection prior to freezing in liquid nitrogen. Datasets were collected at the Canadian Light Source beam-line 08ID at 100 K and were processed using XDS and scaled using XSCALE (36). Using 6MGC as a search model in Phenix Phaser (28), the *E. coli* KpsC-N^2-323^ D160N structure was determined in P3_1_21 at 1.90 Å. After refinement, this structure (KpsC-N_*Ec*_ D160N, with one molecule in the asymmetric unit) was substantially complete; residues 1–320 were traced, and a short, disordered region was evident encompassing 143–146. Again, using 6MGC as a search model in Phenix Phaser (28), the *E. coli* KpsC-N^2-323^ D160C structure was determined in P2_1_ at 1.20 Å resolution. This structure (KpsC-N_*Ec*_ D160C) has two molecules in the asymmetric unit. Chain A has residues 1–323 (plus one residue of the His_6_-tag) traced, with a short, disordered region encompassing 146–147. Chain B has residues 1–323 (plus two residues of the His_6_-tag) traced, with a short, disordered region encompassing 195–201. Data collection and structure refinement statistics are shown for both structures in Table 1. Structure figures were prepared in PyMol v.2.3.4.

### Investigation of the activities of chimeric enzymes from *E. coli* and *T. dismutans* KpsC modules

Reaction mixtures consisted of 50 mM Hepes (pH 8.0), 10 mM MgCl_2_, 0.5 mM Kdo, 1.25 mM CTP, 2 μg *E coli* KdsB, 12 μM acceptors **1** or **3**, and 4 μg of purified Chimera_*Td*_ or Chimera_*Ec*_, in a 20 μl final volume. The concentrations of acceptors **1** and **3** were estimated by measuring the absorbance at 505 nm using an extinction coefficient of 80,000 M^−1^ cm^−1^ (Invitrogen). Reactions were incubated at 37 °C for 30 min. To generate products for subsequent enzyme modification reactions, the reaction volume was doubled, and the mixture was then filtered through Microcon-10 kDa centrifugal filters to remove protein. Ten microliters of the protein-free reaction mixture were then used in 20 μl reactions consisting of 50 mM Hepes (pH 8.0), 10 mM MgCl_2_, 0.5 mM Kdo, 1.25 mM CTP, 2 μg KdsB, and 4 μg of the purified *E. coli* KpsC^2-352^ or KpsC^2-675^ D160A proteins. Reactions were incubated at 37 °C for 30 min. One microliter of the aliquots of each reaction were analyzed by TLC. The TLC plate was developed in freshly prepared ethyl acetate:1-butanol:water:acetic acid mixture (10:8:8:5, v/v), and the products were visualized using a hand-held UV lamp.

### Synthesis of Chimera_*Td*_ reaction product 4 for structural characterization

Reactions were performed in 20 μl volumes to optimize enzyme:acceptor ratios. Reactions contained 50 mM Hepes (pH 8.0), 10 mM MgCl_2_, 1 mM Kdo, 2.5 mM CTP, 1 μg *E coli* KdsB, 0.1 to 1 mM acceptor **1**, and varying quantities of the purified Chimera_*Td*_ protein. After incubation at 37 °C for 30 min, 1 μl aliquots of each reaction were analyzed by TLC as described above. Conversion of acceptor **1** to product **4** was achieved using 0.35 mM acceptor **1**, along with 250 μg Chimera_*Td*_. Once conditions were optimized, the reaction was scaled up to a 500 μl volume incubated at 37 °C for 1 h, with reaction progress being assessed *via* TLC.

Reaction mixture was diluted to 5 ml in water and loaded onto a Sep-Pak Plus C_18_ cartridge previously equilibrated with 10 ml of acetonitrile and 20 ml of water. The column was washed with 20 ml of water and product(s) were eluted in 5 ml of 50% (v/v) acetonitrile/water before pooling the eluent. The eluent was concentrated to 250 μl for further purification *via* size-exclusion chromatography on a Superdex 200 Increase 10/300 Gl column (30 × 1 cm), eluted in 100 mM sodium acetate (at 0.8 ml/min), and monitored using A_503nm_ and TLC. Elution fractions containing product **4** were combined, desalted using Sep-Pak Plus C_18_ cartridge, and dried using a SpeedVac or lyophilization.

### NMR spectroscopy of KpsC *in vitro* reaction product 4

NMR studies were performed at the NMR Centre of the Advanced Analysis Centre at the University of Guelph. The purified reaction product was deuterium-exchanged by freeze-drying twice from 99.9% D_2_O. The sample was suspended in 250 μl of 99.99% D_2_O and placed in a 5 mm Shigemi NMR microtube. ^1^H NMR, and 2D ^1^H-^13^C HSQC spectra were obtained using a Bruker Avance III 600 MHz spectrometer equipped with a 5 mm TCI cryoprobe. Sodium 3-trimethylsilylpropanoate-2,2,3,3-*d*_4_ was added to the sample as a chemical shift reference in the ^1^H and ^13^C dimensions (δ_H_ = 0 ppm, δ_C_ =−1.6 ppm). The two-dimensional ^1^H-^13^C HSQC experiment was collected using standard pulse sequences and parameters.

### Electrospray ionization mass spectrometry analysis of *in vitro* products from KpsC reactions

ESI MS analyses were performed on an Agilent 1260 HPLC liquid chromatograph interfaced with an Agilent UHD 6530 Q-Tof mass spectrometer (Advanced Analysis Centre, University of Guelph). The mass spectrometer electrospray capillary voltage was maintained at 4.0 kV and the drying gas temperature at 250 °C with a flow rate of 8 L min^−1^. Nebulizer pressure was 30 p.s.i., and the fragmentor was set to 160. Nozzle, skimmer, and octopole RF voltages were set at 1000 V, 65 V and 750 V, respectively. Nitrogen (purity > 98%) was used as nebulizing, drying, and collision gas. The mass-to-charge ratio was scanned across the *m/z* range of 50 to 2000 *m/z* in 4 GHz (extended dynamic range) negative-ion modes. Data were collected by target- or data-independent MS/MS acquisition with an MS and MS/MS scan rate of 1.41 spectra s^−1^. The acquisition rate was set at 2 spectra s^−1^. The mass axis was calibrated using the Agilent tuning mix HP0321 (Agilent Technologies) prepared in acetonitrile. Mass spectrometer control, data acquisition, and data analysis were performed with MassHunter Workstation software (https://www.agilent.com/en/product/software-informatics/mass-spectrometry-software). Purified reaction products and *in vitro E. coli* KpsC reaction products with acceptor **1** were separated on a C18 column (Agilent Poroshell 120, EC-C18 50 mm × 3.0 mm, 2.7 μm) with the following solvents: water with 0.1% formic acid (A) and acetonitrile with 0.1% formic acid (B). The mobile phase gradient was as follows: initial conditions were 10% B hold for 1 min; increasing to 100% B in 29 min; column wash at 100% B for 5 min; 20 min re-equilibration. The flow rate was maintained at 0.4 ml min^−1^.

### UV-HPLC analysis of *E. coli* and *A. pleuropneumoniae* KpsC product chain lengths

*In vitro* reactions were performed in 60 μl volumes and contained 50 mM Hepes (pH 8.0), 10 mM MgCl_2_, 3 mM Kdo, 5 mM CTP, 40 μM acceptor **1**, 2.9 μM *E. coli* KdsB, and 6.3 μM of KpsC protein (*E. coli* KpsC^2-675^, *E. coli* KpsC^2-650^, *A. pleuropneumoniae* KpsC^2-689^, and *A. pleuropneumoniae* KpsC^2-670^). The reaction mixtures were incubated at 30 °C. In a time-course experiment, 10 μl aliquots were removed after 1, 10, 30, 60 min, and 21 h and quenched by mixing with 10 μl cold acetonitrile. Precipitated protein was removed by centrifugation at 12,000*g* for 5 min, and supernatant was collected in an HPLC vial. Reaction pellet was then resuspended in 10 μl water before centrifugation at 12,000*g* for 5 min. Supernatant was removed and combined with the supernatant in the HPLC vial for a final volume of 30 μl. HPLC analysis was performed on Agilent 1260 Infinity II LC system equipped with 1260 Infinity II Variable Wavelength Detector. A DNAPac PA-100 column (4 × 50 mm) was used for chromatographic separation with the following solvents: water (A), acetonitrile (B), and 2 M ammonium acetate (C). The mobile phase gradient was as follows: 15 % B was kept constant throughout the run; initial conditions were 0% C; increasing to 75% C in 30 min; hold 75% C for 5 min; decreasing to 0% C in 5 min; 15 min re-equilibration. The flow rate was maintained at 0.5 ml min^−1^, and the column temperature was set to 40 °C. The elution profile was monitored by UV detection at 503 nm and the injection volume was 15 μl. Experiments were repeated three times.

## Data availability

The crystal structures of the KpsC-N_*Ec*_ D160N Kdo adduct and the KpsC-N_*Ec*_ D160C ternary complex are available from the PDB with Accession Numbers 8FUW and 8FUX, respectively. All other data is contained in the manuscript and Supporting information.

## Supporting information

This manuscript includes Supporting information (5, 9, 29).

## Conflict of interest

The authors declare that they have no conflicts of interest with the contents of this article.
