# Supplementary material for: Mechanism and linkage specificities of the dual retaining β-Kdo glycosyltransferase modules of KpsC from bacterial capsule biosynthesis
Source: J Biol Chem. 2023 Mar 15;299(5):104609. doi: 10.1016/j.jbc.2023.104609 (PMC10148158; doi:10.1016/j.jbc.2023.104609)
Supplement: Supplemental data [file mmc1.pdf]

Mechanism and linkage specificities of the dual retaining β-Kdo glycosyltransferase modules of KpsC from bacterial capsule biosynthesis

Liam Doyle[1], Olga G. Ovchinnikova[1], Bo-Shun Huang[2,3], Taylor J.B. Forrester[1], Todd L. Lowary[2,4,5], Matthew S. Kimber[1*], and Chris Whitfield[1*]

[1] Department of Molecular and Cellular Biology, University of Guelph, Guelph, Ontario, Canada N1G 2W1

[2] Department of Chemistry, University of Alberta, Edmonton, Alberta, Canada T6G 2G2

[3] Present address, Dxome CLIA Laboratory Inc., Irvine, California 92618, United States

[4] Institute of Biological Chemistry, Academia Sinica, Nangang, Taipei, Taiwan 11529

[5] Institute of Biochemical Sciences, National Taiwan University, Taipei, Taiwan 106

Materials & Correspondence

*Chris Whitfield, University of Guelph, 50 Stone Road East, Guelph, Ontario N1G 2W1, Canada, Tel: (519)-824-4120 ext. 53361, Email: cwhitfie@uoguelph.ca

*Matthew S. Kimber, University of Guelph, 50 Stone Road East, Guelph, Ontario N1G 2W1, Canada, Tel: (519)-824-4120 ext. 53361, Email: mkimber@uoguelph.ca

Supplementary Results

KpsC-C$_{Td}$ with an active site Asp→Cys substitution forms a stable Kdo adduct *in vivo*

The native KpsC-C$_{Td}$, and a variant substituting Asp165 (of the QXXXD motif) to cysteine, were examined for their ability to form covalent adducts with Kdo. Proteins were overproduced and purified from *E. coli* BL21 and examined using Liquid Chromatography-Mass Spectrometry (LC-MS) The expected molecular weights for KpsC-C$_{Td}$ and KpsC-C$_{Td}$ D165C are 38,225.28 Da and 38,213.33 Da, respectively, reflecting the 11.95 Da mass differences between Asp and Cys. The LC-MS spectrum of KpsC-C$_{Td}$ (**Supplementary Figure 1A**) shows two predominant peaks at 38,225.28 Da and 38,252.57 Da. The former corresponds to the expected mass of the enzyme, whereas the increase in the latter (+27.29) corresponds to the mass increase due to formylation of an amino acid residue (+27.99 Da). Formylmethionine is the first amino acid added during protein synthesis and, while the formyl group is commonly removed by intracellular peptide deformylase enzymes, this process does not always proceed to completion (1). The LC-MS spectrum of KpsC-C$_{Td}$ D165C (**Supplementary Figure 1B**) shows two predominant peaks (38,433.51 Da and 38,461.52 Da), which also appear to differ in the presence of a formyl group (+28.01). However, these peaks also indicate additional increases in mass (+220.18 Da and 220.9 Da, respectively) corresponding to the covalent attachment of a Kdo moiety (+220.18 Da) to KpsC-C$_{Td}$ D165C and *formyl*-KpsC-C$_{Td}$ D165C. Therefore, the KpsC-C$_{Td}$ D165C mass spectrum is consistent with the formation of a stable Kdo adduct.

Crystal structure of KpsC-N$_{Ec}$ D160C variant chain B complex

The structure of KpsC-N$_{Ec}$ D160C chain B superimposes on chain A with an r.m.s.d. of 1.1 Å. Despite binding the acceptor in a very similar fashion, the two chains show key differences in the ligands bound, the position occupied by Kdo-D, and the degree of disorder in active site loops. The acceptor is again found to bind in the active site with full occupancy and low ADPs,

and makes very similar interactions to those seen in chain A. However, in chain B, there is no density visible for the CMP group, other than some very weak residual density modelled as a partially occupied phosphate group. Weak, but clear density extends from the sulfhydryl group of Cys160; this can be modelled as an α-Kdo moiety with partial occupancy (0.72), and with atomic displacement factors much higher than surrounding residues (~40 $Å^2$, compared to ~15 - 25 $Å^2$ for nearby protein atoms) (**Supplementary Figure 3A,B**). Interestingly, Cys160 Cα does not show the shift towards the acceptor as observed in chain A. Chain B's α-Kdo intermediate is significantly reorganized compared to chain A and the D160N structure (**Supplementary Figure 3C,D)**, with the Kdo group rotated ~120° so that it sits largely outside of the usual Kdo-D pocket. In this orientation, the Kdo makes no interactions with the highly conserved Tyr93, Lys133 and Tyr134 side chains; these residues are all 6 Å or more from Kdo-D ((**Supplementary Figure 3C**). Instead, the carboxylate group hydrogen bonds with the side chain hydroxyl and backbone nitrogen of Ser162 and backbone nitrogen of Met161, Arg45 hydrogen bonds with the O7 hydroxyl group, and the O5 hydroxyl group of Kdo-A1 hydrogen bonds with the C1 carboxylate of Kdo A1. This organization places the anomeric carbon 5 Å away from the incoming acceptor nucleophile, the O4 hydroxyl group of the terminal Kdo. Chain B also shows significantly increased disorder in the general region of the active site (**Supplementary Figure 3D**). The region around the Cα3 helix (residues His195-Lys201) is wholly disordered; this region includes residues that contribute to the binding of CMP (His194, Pro195), and stabilizing the α-Kdo intermediate (Asp196). The loop Leu75-Glu80 is also shifted backwards ~3.2 Å away from the active site; this loop stacks on Arg71 and Tyr134, and appears to result in several small, concerted changes in the active site. Interestingly, these motifs are both observed to be disordered in the KpsC-C_{Td} structure, a structure we previously argued to reflect local order-to-disorder transitions in active site loops in the absence of ligands. Finally, the N-terminal domain is shifted to a position intermediate between the apo

structure and chain A. Overall, the structure observed for chain B appears to be partially disorganized and not competent for turnover. The change of Asp160 to Cys160 possibly exerts strain on the protein when attempting to accommodate the adduct. Small differences in crystal packing environment might then allow a structurally relaxed state where residue 160 is unstrained, and active site loops are allowed to adopt their more relaxed, disordered state, at the cost of the favourable interactions mediated by CMP and Kdo-A binding. The positioning of the Kdo outside the binding site then simply reflects the next best interactions available to this group, which is necessarily present as essentially every protein molecule in the crystallization solution has Kdo covalently attached to Cys160.

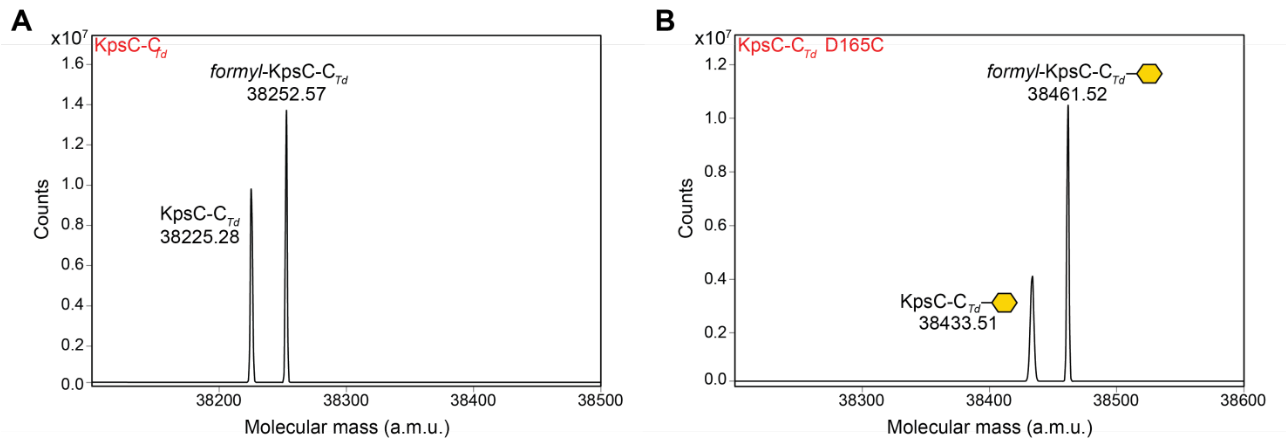

Supplementary Figure 1. LC-MS spectra for KpsC-C*Td* and KpsC-*Td*D165C. *(A)* The wild-type KpsC-C*Td* enzyme shows two peaks of similar abundance with the first corresponding to the expected molecular mass and the second with formylation of an amino acid. (*B*) The KpsC-C*Td* D165C variant also shows two peaks, which differ in formylation of an amino acid, but both species appear to be modified with a Kdo residue. All protein peaks are within 1 a.m.u. of their calculated masses.

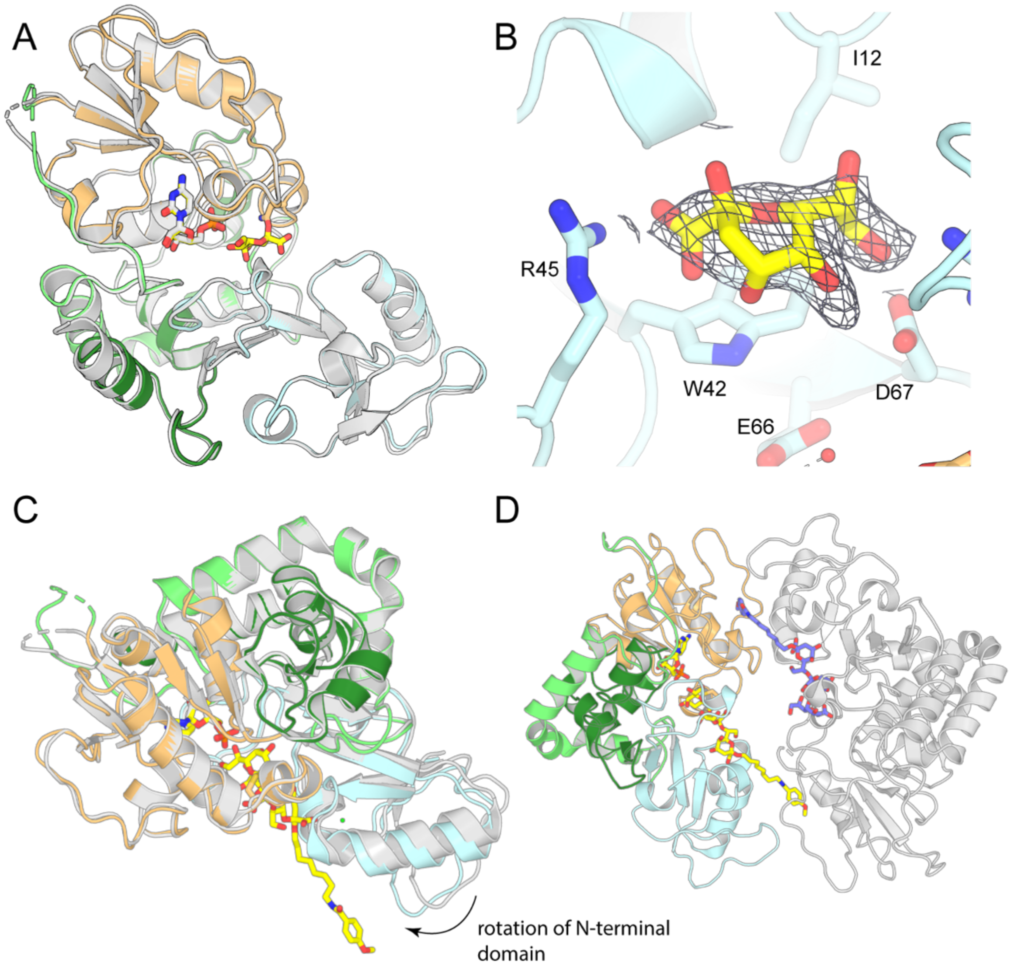

Supplementary Figure 2. Additional structural figures. (*A*) Shows superposition of the KpsC-N$_{Ec}$ D160N Kdo adduct structure (multicoloured) on the KpsC-N$_{Ec}$ CMP complex structure (6MGC). Note that the structure shows minimal shifts. (*B*) Density for the Kdo bound in the Kdo-A2 site in the KpsC-N$_{Ec}$ Kdo adduct structure. 2mFo – DFc electron density is contoured at 0.8 s. (*C*) Superposition of KpsC-N$_{Ec}$ D160C ternary complex structure (multicoloured) on the KpsC-N$_{Ec}$ CMP complex structure (PDB i.d. 6MGC; white). The N-terminal domain rotates inward upon formation of the ternary complex. (*D*) Interactions in the KpsC-N$_{Ec}$ D160C ternary complex structure. Chain A is shown multicoloured with yellow ligands, chain B in white with blue ligands. Note that the two chains adopt a dimer-like arrangement, anchored together by the methoxybenzamide aglycone of the acceptor binding in a non-polar pocket on the opposite chain.

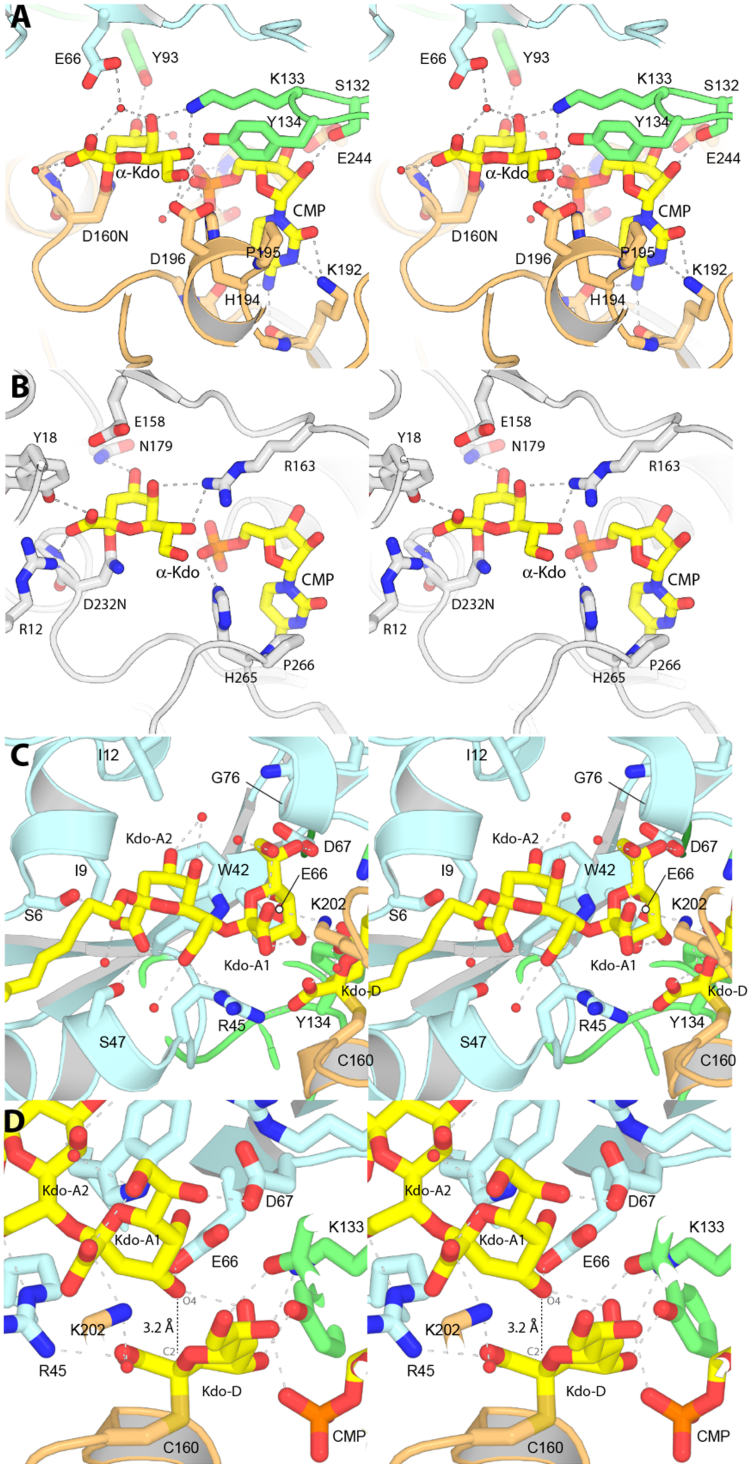

Supplementary Figure 3. Additional stereo structural figures. Details of interactions mediated by the D160N–Kdo adduct are highlighted in (*A*), while (*B*) presents an equivalent view of the structure of $WbbB_{GT99}$ D232N–Kdo adduct complex. Panel (*C*) shows details of interactions mediated by the D160C–Kdo acceptor disaccharide in the active stie of chain A, and (*D*) highlights the organization of the acceptor Kdo-A1 group and the Kdo–C160 adduct.

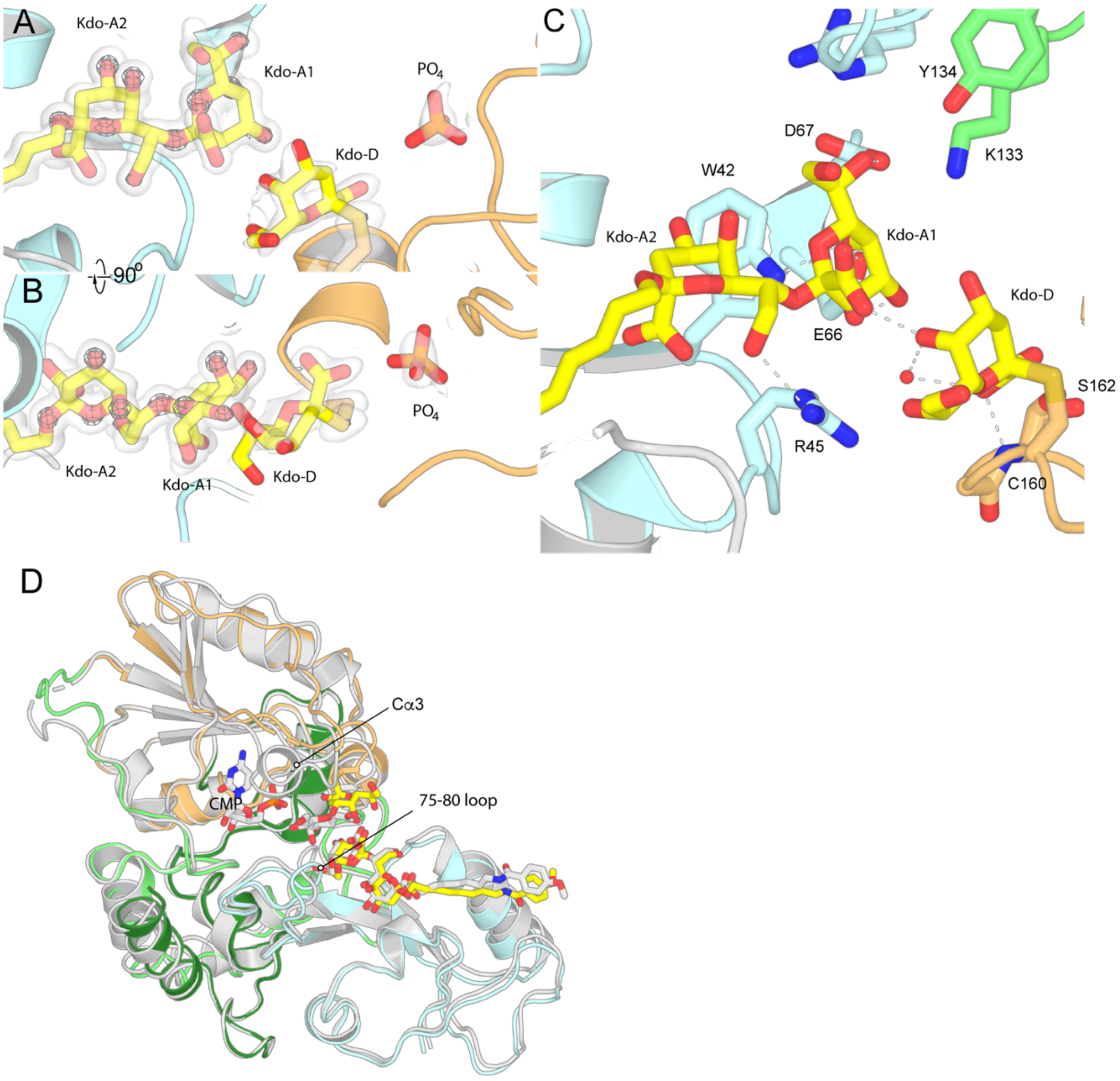

Supplementary Figure 4. Details of ligand binding in chain B of the KpsC-N*Ec* D160C ternary complex structure. (*A*) and (*B*) are orthogonal views showing electron density around the ligands. The transparent white surface shows 2mFo – DFc electron density contoured at 1.5 σ, while the blue mesh shows density at 4 σ. (*C*) Details of ligand interactions in the active site. The acceptor disaccharide makes interactions that are essentially identical to chain A, but the donor is rotated out of its pocket, and appears poorly ordered. Only the phosphate group of CMP appears partially ordered. *(D)* Superposition of chain A (in white) and chain B (multicolored) of the D160C ternary complex.

Supplementary Table 1. ^{1}H and ^{13}C NMR data for the purified enzymatic reaction product 4 (δ, ppm)[1].

Sugar residue		*H-3a (ax)* C-3	*H-3b (eq)*	*H-4* C-4	*H-5* C-5	*H-6* C-6	*H-7* C-7	*H-8a* C-8	*H-8b*
→7)-β-Kdo*p*-(2→	**A**	*1.78*	*2.41*	*3.72*	*4.00*	*3.60*	*4.36*	*3.71*	*3.88*
		36.0		68.7	66.1	73.4	72.2	63.7	
→4)-β-Kdo*p*-(2→	**B**	*1.92*	*2.35*	*3.87*	*4.19*	*3.44*	*3.90*	*3.77*	*3.90*
		34.2		73.5	66.5	74.2	70.5	65.3	
β-Kdo*p*-(2→	**C**	*1.86*	*2.46*	*3.74*	*3.96*	*3.63*	*3.92*	*3.73*	*3.84*
		36.4		68.7	66.6	74.7	70.3	65.6	

[1] ^{1}H-NMR chemical shifts are given in italics.

Supplementary Methods

Supplementary Table 2. Plasmids used in this study.

Plasmid	Description	Source/Ref.
pET28a(+)	Protein expression vector under control of T7 promoter; Kn^r	Novagen
pWQ879	pET28a(+) derivative expressing *E. coli* $KpsC^{2\text{-}675}$	(2)
pWQ880	pET28a(+) derivative expressing *E. coli* $KpsN^{2\text{-}352}$	(2)
pWQ886	pET28a(+) derivative expressing *E. coli* $KpsC^{2\text{-}675}$ D160A	(2)
pWQ963	pET28a(+) derivative expressing *E. coli* $KdsB^{2\text{-}248}$	(3)
pWQ965	pET28a(+) derivative expressing *T. dismutans* KpsC-$N^{1\text{-}318}$	(3)
pWQ966	pET28a(+) derivative expressing *T. dismutans* KpsC-$C^{1\text{-}326}$	(3)
pWQ1116	pET28a(+) derivative expressing *E. coli* $KpsC^{2\text{-}650}$	This study
pWQ1117	pET28a(+) derivative expressing *T. dismutans* KpsC-$C^{1\text{-}326}$ D165C	This study
pWQ1118	pET28a(+) derivative expressing *T. dismutans* KpsC chimera (*T. dismutans* KpsC-$N^{1\text{-}68}$ + *T. dismutans* KpsC-$C^{74\text{-}326}$ chimera)	This study
pWQ1119	pET28a(+) derivative expressing *E. coli* KpsC-$N^{2\text{-}323}$	This study
pWQ1120	pET28a(+) derivative expressing *E. coli* KpsC chimera (*E. coli* KpsC-$N^{2\text{-}69}$ + *T. dismutans* KpsC-$C^{74\text{-}326}$ chimera)	This study
pWQ1121	pET28a(+) derivative expressing *E. coli* KpsC-$N^{2\text{-}323}$ D160C	This study
pWQ1122	pET28a(+) derivative expressing *E. coli* KpsC-$N^{2\text{-}323}$ D160N	This study
pWQ1123	pET28a(+) derivative expressing *E. coli* KpsC-$N^{2\text{-}323}$ D160A	This study
pWQ1124	pET28a(+) derivative expressing *A. pleuropneumoniae* $KpsC^{2\text{-}689}$	This study
pWQ1125	pET28a(+) derivative expressing *A. pleuropneumoniae* $KpsC^{2\text{-}670}$	This study

Supplementary Table 3. Sequences of oligonucleotide primers.

Plasmid	Primer	Sequence[a,b] (5′→3′)
pLD18, pLD71	OL1034	gatcccatgggcATTGGCATTTACTCGCCTGG
pLD18	1030-LD	catgctcgagttagtggtggtggtggtggtgCATCGGCTTGCGCGGTGTCTGGAT
pLD60	1781-LD	GTTCCTGGTCAGGTGGAAAGTtgtGCAAGCATCAAATTTGG
pLD60	1782-LD	GGCTACCAAATTTGATGCTTGCacaACTTTCCACCTGACC
pLD64	1928-LD	ctttaagaaggagatataccATGCCGGTTGGTGTTTTAGCCGT
pLD64, pLD77	1929-LD	ggtggtggtggtgctcgagCGGCAGGTTTTTTTCAGGTTCAC
pLD64	1930-LD	AGACCAAtGCTACGaAtAAAACCATCTTCCAGTGCCAGATACGG
pLD64	1931-LD	GGTTTTATTCGTAGCATTGGTCTG
pLD71	2076-LD	gtcactcgagttaATGTCGACGTTGTAATTGCAGCCA
pLD77	1932-LD	ctttaagaaggagatataccATGGGCATTGGCATTTACTCGCCTGG
pLD77	2277-LD	GAAGATGGTTTTATTCGTAGCATTG
pLD77	2278-LD	CaAtgctACGaAtAAAaCCATCTTCCAGACGAATGACGGGTTTTCC
pLD81	2315-LD	CATCGTTCTGGTTGTCGATCAGACATTTAATtgTATGTCAGTGACGTATG
pLD81	2316-LD	CATACGTCACTGACATAcaATTAAATGTCTGATCGACAACCAGAACGATG
pLD82	2313-LD	GGTTGTCGATCAGACATTTAATaATATGTCAGTGACGTATGG
pLD82	2314-LD	CCATACGTCACTGACATATtATTAAATGTCTGATCGACAACC

pLD103	OL1099	GCCATACGTCACTGACATAgCATTAAATGTCTGATCGAC
pLD103	OL1100	GTCGATCAGACATTTAATGcTATGTCAGTGACGTATGGC
pOL202, pOL207	2193-CN	gatcccATGGCACAGTGTAGCATTACC
pOL202	2194-CN	gatcctcgagCTGCAGGGTACGATACAGC
pOL207	2463-CN	gatcctcgagTTTAATACCGCTATGGCTCAG

[a]Restriction sites are underlined.
[b]Sequences complementary to the template are upper-case.

Experimental Procedures for the Synthesis of 2 and 3. The reaction schemes for synthesis are shown in (**Supplementary Figure 5**)

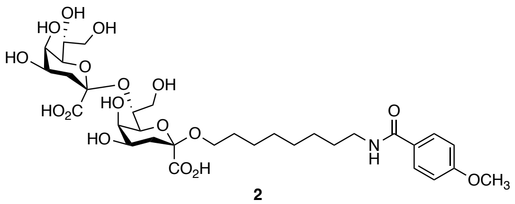

2

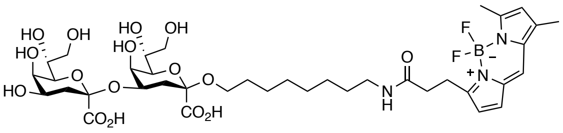

3

Methyl (8-Azidooctyl 8-*O*-*tert*-butyldiphenylsilyl-3-deoxy-4,5-*O*-isopropylidene-β-D-*manno*-2-octulopyranosid)-onate (**S2**). To a solution of **S1** (2) (270 mg, 0.67 mmol) in DMF (6.7 mL) was added imidazole (68.0 mg, 1.00 mmol) and TBDPSCl (225 μL, 238 mg, 0.87 mmol) at 0 ºC. The reaction mixture was warmed to room temperature and after 16 h the excess TBDPSCl was quenched by the addition of CH_3OH. The reaction mixture was concentrated and then diluted with EtOAc and washed with H_2O. The resulting organic layer was washed with satd. aq. $NaHCO_3$, dried with $MgSO_4$, filtered, and concentrated under reduced pressure. The resulting residue was then dissolved in acetonitrile (6.7 mL) and 10-camphorsulfonic acid (47 mg, 0.201 mmol) and 2,2-dimethoxypropane (107 μL, 91 mg, 0.87 mmol) were added at room temperature. After 1 h, triethylamine was added. The reaction mixture was concentrated and the resulting residue was purified by column chromatography (4:1 hexane–EtOAc) to give **S2** (366 mg, 80%) as a colorless oil. $[\alpha]^{25}_{D}$ +15.9 (*c* 0.31, $CHCl_3$); R_f 0.24 (4:1 hexane–EtOAc); ^1H NMR ($CDCl_3$, 500 MHz) δ_H 7.74–7.68 (m, 4H, ArH), 7.46–7.42 (m, 2H, ArH), 7.42–7.36 (m, 4H, ArH), 4.43 (dt, 1H, $J_{4,3a}$ = $J_{4,3b}$ = 5.0 Hz, $J_{4,5}$ = 7.0 Hz, H-4), 4.32 (dd, 1H, $J_{5,4}$ = 7.0 Hz, $J_{5,6}$ = 2.0 Hz, H-5), 4.06 (ddd, 1H, $J_{7,6}$ = 8.0 Hz, $J_{7,8a}$ = 4.0 Hz, $J_{7,8b}$ = 6.5 Hz, H-7), 4.00 (dd, 1H, $J_{8a,7}$ = 4.0, J_{gem} = 10.5 Hz, H-8a), 3.84 (dd, 1H, $J_{8b,7}$ = 6.5 Hz, J_{gem} = 10.5 Hz, H-8b), 3.69 (dd, $J_{6,5}$ = 2.0 Hz, $J_{6,7}$ = 8.0 Hz, 1H, H-6),

3.65 (s, 3H, COOC*H*$_3$), 3.58 (dt, 1H, *J* = 9.5, 6.5 Hz, octyl OC*H*$_2$), 3.27–3.24 (m, 3H, octyl OC*H*$_2$, C*H*$_2$N$_3$), 2.10 (d, 2H, *J* = 5.0 Hz, H-3a, H-3b), 1.62–1.55 (m, 4H, octyl C*H*$_2$), 1.51 (s, 3H, C(C*H*$_3$)$_2$), 1.36 (s, 3H, C(C*H*$_3$)$_2$), 1.36–1.27 (m, 8H, octyl C*H*$_2$), 1.09 (s, 9H, C(C*H*$_3$)$_3$); ^{13}C NMR (CDCl$_3$, 125 MHz) δ_C 170.1 (C-1), 135.6 (Ar), 133.3 (Ar), 133.2 (Ar), 129.8 (Ar), 129.7 (Ar), 127.7 (Ar), 109.2((CH$_3$)$_2$*C*), 98.5 (C-2), 72.0 (C-6), 71.1 (C-5), 70.7 (C-7), 70.5 (C-4), 65.4 (C-8), 64.2 (octyl O*C*H$_2$), 52.1 (COO*C*H$_3$), 51.5 (*C*H$_2$N$_3$), 33.8 (C-3), 29.7 (octyl *C*H$_2$), 29.2 (octyl *C*H$_2$), 29.1 (octyl *C*H$_2$), 28.8 (octyl *C*H$_2$), 27.0 (octyl *C*H$_2$), 26.9 ((*C*H$_3$)$_2$C), 26.7 ((*C*H$_3$)$_2$C), 26.5 ((*C*H$_3$)$_3$C), 26.4 (octyl *C*H$_2$), 19.3 (CH$_3$)$_3$*C*); HRMS (ESI) calcd. for $C_{36}H_{53}N_3NaO_8Si$ [M+Na]$^+$ 706.3494; found 706.3490.

Methyl {(8-Azidooctyl 8-*O*-*tert*-butyldiphenyl-3-deoxy-7-*O*-[methyl 3-deoxy-β-D-*manno*-2-octulopyranosyl]onate)-β-D-*manno*-2-octulopyranosid}onate (**S5**). A mixture of **S2** (81.5 mg, 0.175 mmol) and thioglycoside **S3** (2) (30.0 mg, 0.0439 mmol) and 4Å molecular sieves (100 mg) in CH_2Cl_2–CH_3CN (2:3, 1.5 mL) was stirred under an Ar atmosphere at room temperature for 30 min. The mixture was then cooled to –70 °C and then NIS (79.0 mg, 0.351 mmol) and TfOH (0.39 μL, 0.66 mg, 4.39 μmol) were added. After stirring at –70 °C for 1 h, trimethylamine was added to the reaction mixture. The reaction was warmed to room temperature and then a small amount of H_2O was added, followed by solid $Na_2S_2O_3 \cdot 5H_2O$ until the solution was colorless. The mixture was dried with $MgSO_4$ and then filtered and concentrated under reduced pressure. The resulting residue was purified by column chromatography (4:1, hexane–EtOAc) to give a mixture of **S4** and a glycal by-product formed by elimination of the **S3**. To a solution of this mixture (19.5 mg, 0.018 mmol) in CH_3OH (1.0 mL) was added sodium methoxide (0.1 mg, 1.8 μmol) at room temperature. After 24 h, the reaction mixture was neutralized by the addition of Amberlite IR-120 H$^+$ resin. The mixture was filtered and the filtrate concentrated under reduced pressure. The residue was triturated with EtOAc to remove most of the deacetylated glycal that was formed in the previous

glycosylation step. The deacylated glycal is insoluble in EtOAc. The solution was filtered and the filtrate was concentrated under reduced pressure. The residue was then dissolved in CH_3OH (2.0 mL) and treated with 1N HCl (0.5 mL) at room temperature. After 24 h, the reaction mixture was diluted with CH_2Cl_2 and washed with satd. aq. $NaHCO_3$ and H_2O. The organic layer was then dried with $MgSO_4$ and filtered. The filtrate was concentrated under reduced pressure and the residue was purified by column chromatography (1:9 CH_3OH–EtOAc) to give **S5** (10.1 mg, 64%) as a colorless oil. $[\alpha]^{25}_D$ +21.8 (*c* 0.17, CH_3OH); R_f 0.17 (1:9 CH_3OH–EtOAc); ^{1}H NMR (CD_3OD, 500 MHz) δ_H 7.74–7.69 (m, 4H, ArH), 7.46–7.32 (m, 6H, ArH), 4.37 (dt, 1H, $J_{7,6}$ = 8.0 Hz, $J_{7,8a}$ = $J_{7,8b}$ = 2.5 Hz, H-7), 4.15 (d, 1H, $J_{6,7}$ = 8.0 Hz, H-6), 4.01 (d, 1H, $J_{5,4}$ = 3.0 Hz, H-5), 3.94–3.83 (m, 3H, H-8a, H-6', H-7'), 3.79–3.75 (m, 1H, H-8'a), 3.72–3.64 (m, 3H, H-4, H-8b, octyl OCH_2), 3.63 (s, 3H, COOCH_3), 3.62–3.54 (m, 2H, H-4', H-8'b), 3.40-3.35 (m, 5H, H-5', COOCH_3, octyl OCH_2), 3.23 (t, 2H, J = 7.0 Hz, CH_2N_3), 2.47 (dd, 1H, J = 12.5, 4.5 Hz, H-3_{eq}'), 2.18 (dd, 1H, J = 12.5, 4.5 Hz, H-3_{eq}), 2.09 (t, 1H, J = 12.5 Hz, H-3_{ax}'), 2.03 (t, 1H, J = 12.5 Hz, H-3_{ax}), 1.62–1.44 (m, 4H, octyl CH_2), 1.38–1.22 (m, 8H, octyl CH_2), 1.05 (s, 9H, C(CH_3)$_3$); ^{13}C NMR (CD_3OD, 125 MHz) δ_C 171.0 (C-1), 170.0 (C-1'), 137.0 (Ar), 136.9 (Ar), 135.1 (Ar), 134.7 (Ar), 130.8 (Ar), 130.7 (Ar), 128.8 (Ar), 128.6 (Ar), 100.9 (C-2), 100.5 (C-2'), 76.1 (C-5'), 74.7 (C-6), 74.4 (C-7), 71.7 (C-7'), 68.4 (C-4'), 68.2 (C-4), 67.21 (C-5), 67.17 (C-6'), 65.07 (C-8'), 65.00 (octyl OCH_2), 64.0 (C-8), 53.0 (COO*C*H_3), 53.7 (COO*C*H_3), 52.4 (CH_2N_3), 36.4 (C-3), 35.8 (C-3'), 30.8 (octyl *C*H_2), 30.22 (octyl *C*H_2), 30.19 (octyl *C*H_2), 29.9 (octyl *C*H_2), 27.8 (octyl *C*H_2), 27.5 (octyl *C*H_2), 26.9 (C(*C*H_3)$_3$), 20.3 (*C*(CH_3)$_3$); HRMS (ESI) calcd. for $C_{42}H_{63}N_3NaO_{15}Si$ $[M+Na]^+$ 900.3920; found 900.3912.

[8-Aminooctyl 3-deoxy-7-*O*-(3-deoxy-β-D-*manno*-2-octulopyranosyl)onic acid]-β-D-*manno*-2-octulopyranosid]onic acid (S6). To a solution of **S5** (10.1 mg, 0.011 mmol) in H_2O (1 mL) was added 1N NaOH (0.5 mL) at room temperature. After 24 h, the reaction mixture was neutralized

with the addition of Amberlite IR-120 H^+ resin. The mixture was filtered and the filtrate concentrated under reduced pressure. The residue was then dissolved in H_2O (1 mL) and Pd/C was added (14 mg) at room temperature. The flask was then flushed with H_2 gas and the mixture was stirred 1 h under H_2 atmosphere at room temperature. After 1 h, the reaction mixture was filtered through Celite to remove Pd/C and the filtrate was concentrated under reduced pressure. The resulting residue was purified by column chromatography (3:2:1 EtOAc–CH_3OH–H_2O) followed by size exclusion column chromatography (LH-20) using the eluant (1:1, CH_3OH–H_2O) to give **S6** (5.5 mg, 84%) as a colorless oil. $[\alpha]^{25}_D$ +27.1 (*c* 0.3, H_2O); R_f 0.24 (3:2:1:0.5 EtOAc–CH_3OH–H_2O–AcOH); ^1H NMR (D_2O, 500 MHz) δ_H 4.47–4.43 (m, 1H, H-7), 4.07 (d, 1H, $J_{5,4}$ = 2.9 Hz, H-5), 4.04–3.97 (m, 3H, H-5', H-7', H-8'a), 3.94 (dd, 1H, $J_{8a,7}$ = 2.9 Hz, J_{gem} = 13.1 Hz, H-8a), 3.85–3.72 (m, 5H, H-4, H-8b, H-4', H-8'b, octyl OCH_2), 3.66 (d, 1H, $J_{6,7}$ = 9.1 Hz, H-6), 3.52 (d, 1H, *J* = 9.1 Hz, H-6'), 3.48 (dt, 1H, *J* = 9.4, 6.8 Hz, octyl OCH_2), 3.04 (t, 2H, *J* = 7.7 Hz, CH_2N), 2.49 (dd, 1H, *J* = 12.1, 4.7 Hz, H-3_{eq}'), 2.15 (dd, 1H, *J* = 12.0, 4.6 Hz, H-3_{eq}), 1.88 (app t, 1H, *J* = 12.1 Hz, H-3_{ax}'), 1.84 (app t, 1H, *J* = 12.0 Hz, H-3_{ax}), 1.74–1.66 (m, 2H, octyl CH_2), 1.63–1.55 (m, 2H, octyl CH_2), 1.46–1.33 (m, 8H, octyl CH_2); ^{13}C NMR (D_2O, 125 MHz) δ_C 174.99 (C-1, $J_{C1,H3ax}$ = 5.5 Hz), 174.94 (C-1', $J_{C1',H3'ax}$ = 5.8 Hz), 102.0 (C-2), 101.3 (C-2'), 74.4 (C-6'), 73.1 (C-6), 72.1 (C-7), 70.2 (C-7'), 68.50 (C-4), 68.46 (C-4'), 66.3 (C-5'), 65.9 (C-5), 65.7 (octyl OCH_2), 65.1 (C-8'), 63.0 (C-8), 40.5 (CH_2N), 35.7 (C-3), 35.4 (C-3'), 29.7 (octyl CH_2), 28.84 (octyl CH_2), 28.78 (octyl CH_2), 27.6 (octyl CH_2), 26.3 (octyl CH_2), 25.8 (octyl CH_2); HRMS (ESI) calcd. for $C_{24}H_{42}NO_{15}$ $[M–H]^-$ 584.2560; found 584.2559.

[8-Aniosoylamidooctyl 3-deoxy-7-*O*-(3-deoxy-β-D-*manno*-2-octulopyranosyl)onic acid]-β-D-*manno*-2-octulopyranosid]onic acid (2). To a solution of **S6** (10.0 mg, 0.0171 mmol) in H_2O (1 mL) was added potassium carbonate (9.4 mg, 0.0683 mmol) and *p*-anisoyl chloride (4.4 mg, 0.0256 mmol) at room temperature. After stirring for 24 h, the reaction mixture was concentrated

under reduced pressure. The resulting residue was purified by column chromatography (5:2:1 EtOAc–CH_3OH–H_2O) followed by size exclusion column chromatography (LH-20) using the eluant (1:1, CH_3OH–H_2O) to give **2** (10.2 mg, 83%) as an amorphous solid. $[\alpha]^{25}_{D}$ +10.6 (*c* 0.1, H_2O); R_f 0.15 (5:2:1:0.5 EtOAc–CH_3OH–H_2O–AcOH); ^{1}H NMR (D_2O, 700 MHz) δ_H 7.78 (d, 2H, *J* = 8.9 Hz, ArH), 7.11 (d, 2H, *J* = 8.9 Hz, ArH), 4.45–4.43 (m, 1H, H-7), 4.05 (d, 1H, *J* = 2.6 Hz, H-5), 4.01–3.97 (m, 3H, H-5', H-7', H-8'a), 3.94–3.91 (m, 4H, OCH_3, H-8a), 3.85–3.70 (m, 5H, H-4, H-8b, H-4', H-8'b, OCH_2), 3.65 (d, 1H, *J* = 9.4 Hz, H-6), 3.51 (d, 1H, *J* = 8.8 Hz, H-6'), 3.45 (dt, 1H, *J* = 9.1, 6.9 Hz, OCH_2), 3.40 (t, 2H, *J* = 7.7 Hz, CH_2N), 2.48 (dd, 1H, *J* = 12.1, 4.6 Hz, H-3_{eq}'), 2.46 (dd, 1H, *J* = 12.1, 4.6 Hz, H-3_{eq}), 1.90 (app t, 1H, *J* = 12.1 Hz, H-3_{ax}'), 1.83 (app t, 1H, *J* = 12.1 Hz, H-3_{ax}), 1.67–1.61 (m, 2H, octyl CH_2), 1.60–1.54 (m, 2H, octyl CH_2), 1.44–1.31 (m, 8H, octyl CH_2); ^{13}C NMR (D_2O, 125 MHz) δ_C 174.88 (C-1 or C-1'), 174.81 (C-1' or C-1), 171.0 (C=O), 162.6 (Ar), 129.8 (Ar), 127.3 (Ar), 114.9 (Ar), 101.9 (C-2), 101.3 (C-2'), 74.3 (C-6'), 73.0 (C-6), 71.9 (C-7), 70.1 (C-7'), 68.40 (C-4 or C-4'), 68.36 (C-4' or C-4), 66.2 (C-5'), 65.78 (C-5), 65.71 (octyl OCH_2), 64.9 (C-8'), 63.0 (C-8), 56.4 (O*C*H_3), 40.8 (*C*H_2N), 35.6 (C-3), 35.1 (C-3'), 29.8 (octyl *C*H_2), 29.18 (octyl *C*H_2), 29.14 (octyl *C*H_2), 29.11 (octyl *C*H_2), 26.9 (octyl *C*H_2), 25.9 (octyl *C*H_2); HRMS (ESI) calcd. for $C_{32}H_{48}NO_{17}$ $[M–H]^-$ 718.2928; found 718.2915.

8-aminooctyl 3-deoxy-β-D-*manno*-2-octulopyranosidonic acid, BODIPY conjugate (S8). To a solution of **S7** (2) (9.4 mg, 25.7 μmol) in H_2O (1.0 mL) and DMF (0.05 mL) at 0 °C was added potassium bicarbonate (10.7 mg, 0077 mmol) and BODIPY-NHS ester (10.0 mg, 25.7 μmol). After stirring at 0 °C for 2 h, the reaction mixture was concentrated under reduced pressure. The resulting residue was purified by column chromatography (7:2:1, EtOAc–CH_3OH–H_2O) and size exclusion chromatography (Sephadex LH-20, 1:1, CH_2Cl_2–CH_3OH) to give **S8** (10.1 mg, 61%) as a blue amorphous solid. $[\alpha]^{25}_{D}$ +1.6 (*c* 0.1, H_2O); R_f 0.20 (7:2:1:0.5, EtOAc–CH_3OH–H_2O–HOAc) ^{1}H NMR (D_2O, 700 MHz) δ 7.58 (s, 1H, ArH), 7.17 (d, 1H, *J* = 4.0 Hz, ArH), 6.49 (d, 1H, *J* = 4.0

Hz, ArH), 6.42 (s, 1H, ArH), 4.07–3.97 (m, 3H, H-5, H-7, H-8a), 3.88–3.70 (m, 4H, H-4, H-6, H-8b, OC*H*$_2$), 3.50–3.41 (m, 1H, OC*H*$_2$), 3.30 (app t, 2H, *J* = 6.8 Hz, C*H*$_2$), 3.19 (app t, 2H, *J* = 6.4 Hz, C*H*$_2$N), 2.77 (app t, 2H, *J* = 6.8 Hz, C*H*$_2$), 2.62 (s, 3H, C*H*$_3$), 2.52 (dd, 1H, *J* = 12.2, 4.6 Hz, H-3$_{eq}$), 2.37 (s, 3H, C*H*$_3$), 1.87 (app t, 1H, *J* = 12.2 Hz, H-3$_{ax}$), 1.59–1.05 (m, 12H, octyl C*H*$_2$); ^{13}C NMR (D_2O, 125 MHz) δ 175.5 (C=O), 174.8 (C=O), 162.4 (Ar), 156.5 (Ar), 147.4 (Ar), 136.3 (Ar), 134.2 (Ar), 129.7 (Ar), 125.8 (Ar), 121.6 (Ar), 122.0 (Ar), 117.8 (Ar), 102.1, 74.2 (C-6), 70.0 (C-7), 68.5 (C-4), 66.3 (C-5), 65.8 (O*C*H$_2$), 65.1 (C-8), 40.2 (*C*H$_2$), 35.8 (C-3), 30.0 (*C*H$_2$), 29.5 (octyl *C*H$_2$), 29.4 (octyl *C*H$_2$), 29.2 (octyl *C*H$_2$), 26.8 (octyl *C*H$_2$), 26.2 (octyl *C*H$_2$), 25.5 (octyl *C*H$_2$), 15.2 (*C*H$_3$), 11.5 (*C*H$_3$); HRMS (ESI) calcd. for $C_{30}H_{43}$[11B]$F_2N_3O_9$ [M–H]$^-$ 638.3066 found 638.3087.

8-aminooctyl [3-deoxy-4-*O*-(3-deoxy-β-D-*manno*-2-octulopyranosyl)onic acid]-β-D-*manno*-2-octulopyranosid]onic acid, BODIPY conjugate (3). Compound **3** was synthesized enzymatically from **S8**, using *E. coli* KpsC-N (KpsC$^{2\text{-}252}$ encoded by plasmid pWQ880) and reaction conditions reported previously (2).

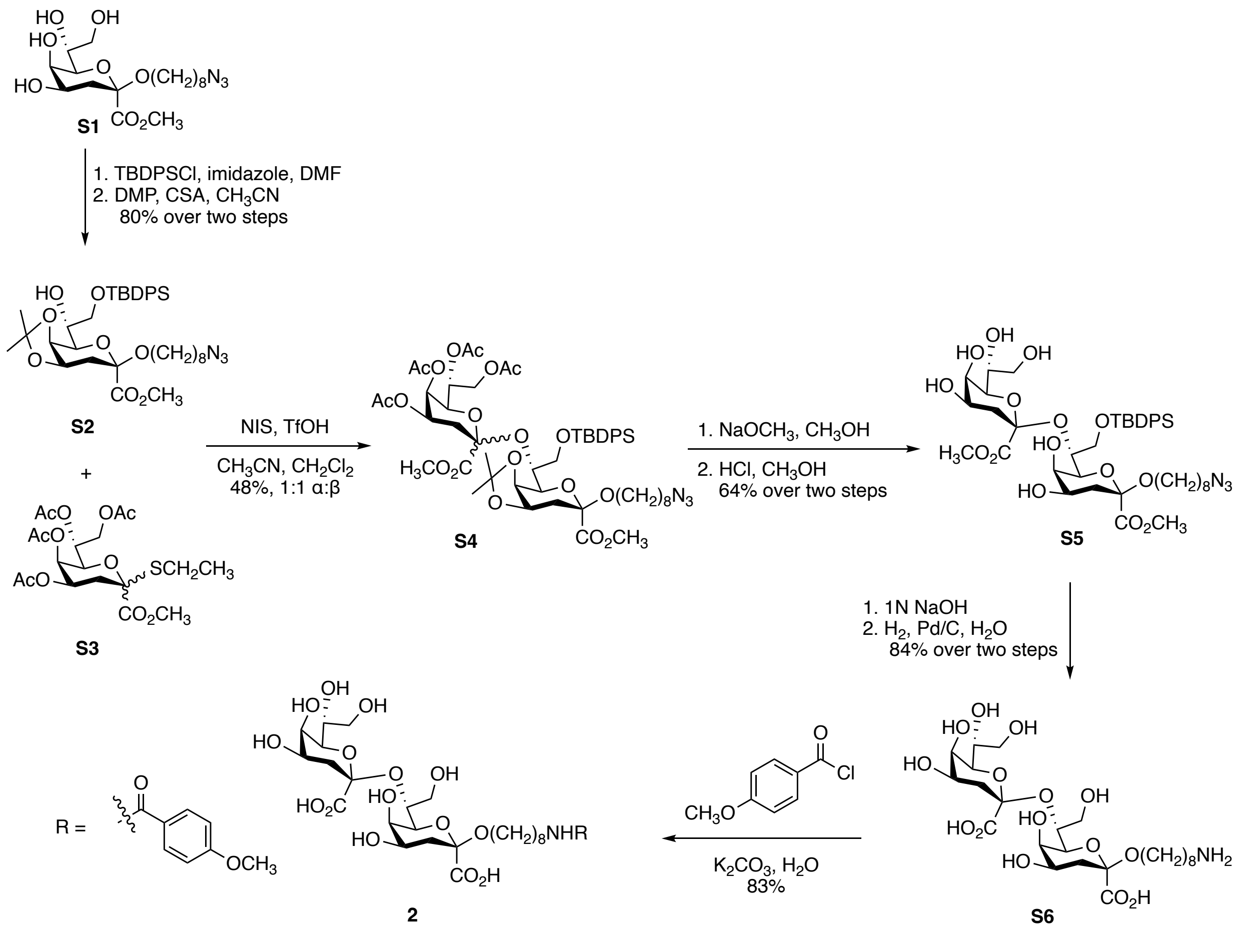

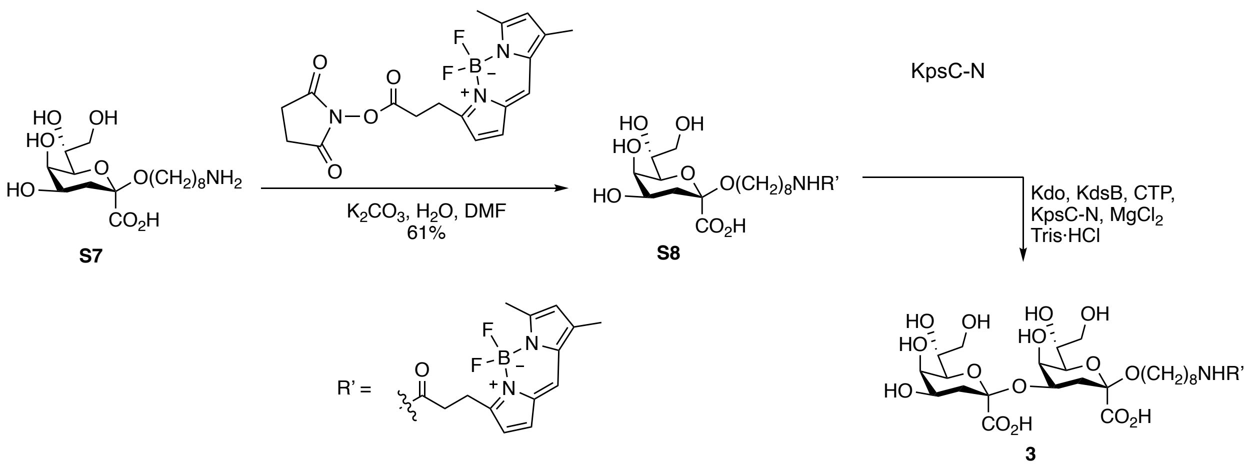

Supplementary Figure 5. Reaction schemes for chemical and enzymatic synthesis of compounds 2 and 3.

References:

1. N. Gupta, *et al.*, Whole proteome analysis of post-translational modifications: Applications of mass-spectrometry for proteogenomic annotation. *Genome Res* 17, 1362–1377 (2007).

2. O. G. Ovchinnikova, *et al.*, Biochemical characterization of bifunctional 3-deoxy-β-D-manno-oct-2-ulosonic acid (β-Kdo) transferase KpsC from *Escherichia coli* involved in capsule biosynthesis. *J Biol Chem* 291, 21519–21530 (2016).

3. L. Doyle, *et al.*, Biosynthesis of a conserved glycolipid anchor for Gram-negative bacterial capsules. *Nat Chem Biol* 15, 632–640 (2019).
